# Comparative neuronal morphology of the cerebellar cortex in afrotherians, carnivores, cetartiodactyls, and primates

**DOI:** 10.3389/fnana.2014.00024

**Published:** 2014-04-23

**Authors:** Bob Jacobs, Nicholas L. Johnson, Devin Wahl, Matthew Schall, Busisiwe C. Maseko, Albert Lewandowski, Mary A. Raghanti, Bridget Wicinski, Camilla Butti, William D. Hopkins, Mads F. Bertelsen, Timothy Walsh, John R. Roberts, Roger L. Reep, Patrick R. Hof, Chet C. Sherwood, Paul R. Manger

**Affiliations:** ^1^Laboratory of Quantitative Neuromorphology, Psychology, Colorado CollegeColorado Springs, CO, USA; ^2^Faculty of Health Sciences, School of Anatomical Sciences, University of the WitwatersrandJohannesburg, South Africa; ^3^Cleveland Metroparks ZooCleveland, OH, USA; ^4^Department of Anthropology, Kent State UniversityKent, OH, USA; ^5^Fishberg Department of Neuroscience and Friedman Brain Institute, Icahn School of Medicine at Mount SinaiNew York, NY, USA; ^6^Division of Developmental and Cognitive Neuroscience, Yerkes National Primate Research CenterAtlanta, GA, USA; ^7^Center for Zoo and Wild Animal Health, Copenhagen ZooFrederiksberg, Denmark; ^8^Smithsonian National Zoological ParkWashington, DC, USA; ^9^Department of Physiological Sciences, University of FloridaGainesville, FL, USA; ^10^Department of Anthropology, The George Washington UniversityWashington, DC, USA

**Keywords:** dendrite, morphometry, Golgi method, brain evolution, cerebellum

## Abstract

Although the basic morphological characteristics of neurons in the cerebellar cortex have been documented in several species, virtually nothing is known about the quantitative morphological characteristics of these neurons across different taxa. To that end, the present study investigated cerebellar neuronal morphology among eight different, large-brained mammalian species comprising a broad phylogenetic range: afrotherians (African elephant, Florida manatee), carnivores (Siberian tiger, clouded leopard), cetartiodactyls (humpback whale, giraffe) and primates (human, common chimpanzee). Specifically, several neuron types (e.g., stellate, basket, Lugaro, Golgi, and granule neurons; *N* = 317) of the cerebellar cortex were stained with a modified rapid Golgi technique and quantified on a computer-assisted microscopy system. There was a 64-fold variation in brain mass across species in our sample (from clouded leopard to the elephant) and a 103-fold variation in cerebellar volume. Most dendritic measures tended to increase with cerebellar volume. The cerebellar cortex in these species exhibited the trilaminate pattern common to all mammals. Morphologically, neuron types in the cerebellar cortex were generally consistent with those described in primates (Fox et al., 1967) and rodents (Palay and Chan-Palay, 1974), although there was substantial quantitative variation across species. In particular, Lugaro neurons in the elephant appeared to be disproportionately larger than those in other species. To explore potential quantitative differences in dendritic measures across species, MARSplines analyses were used to evaluate whether species could be differentiated from each other based on dendritic characteristics alone. Results of these analyses indicated that there were significant differences among all species in dendritic measures.

## Introduction

In terms of gross anatomy, the cerebellum appears to have a common plan in all mammals (Bolk, 1906; Breathnach, 1955; Larsell, 1970; Sultan and Braitenberg, 1993), although absolute and relative size can vary considerably (Marino et al., 2000; Maseko et al., 2012b). Histologically, cerebellar cortex exhibits a generally trilaminate architecture, which is similar in birds and mammals (Ramón y Cajal, 1909, 1911; Iwaniuk et al., 2006; Sultan and Glickstein, 2007). Whereas limited aspects of cerebellar neuron morphology have been described in some vertebrate species (e.g., mormyrid electric fish: Han et al., 2006; teleost fish: Murakami and Morita, 1987; alligator: Nicholson and Llinas, 1971; cat: Melik-Musyan and Fanardzhyan, 2004; duck: O'Leary et al., 1968; dolphin: Adanina, 1965; rhesus monkey: Fox et al., 1967; Rakic, 1972; human: Braak and Braak, 1983), the most detailed research has focused on rodents. In particular, Palay and Chan-Palay (1974) provided a comprehensive examination of the cerebellar cortex of the rat, documenting organizational features, neuronal morphology, and ultrastructure at the electron microscopic level. Recently, we expanded the scope of such investigations with an examination of neuronal morphology in the cerebellar cortex of the African elephant (Maseko et al., 2012a). The current, rapid Golgi study is part of a larger project to document neuronal morphology of both the cerebral neocortex (Jacobs et al., 2011) and the cerebellar cortex in large brained mammals not previously examined. Such comparative investigations may help discern which aspects of neuronal morphology are general to all vertebrates, and which are specific to particular species (Meek et al., 2008). To this end, we examine cortical neuronal morphology in the cerebella of eight different mammalian species comprising four diverse taxa: afrotherians (African elephant, Florida manatee), carnivores (Siberian tiger, clouded leopard), cetartiodactyls (humpback whale, giraffe), and primates (human, common chimpanzee).

Although there are many representative freehand and camera lucida drawings of cerebellar cortex neurons (Ramón y Cajal, 1909, 1911; Chan-Palay and Palay, 1970, 1972; Palay and Chan-Palay, 1974; Braak and Braak, 1983; Bishop, 1993; Lainé and Axelrad, 1996), very few cerebellar neurons have been digitally reconstructed relative to those in the neocortex and hippocampus (Halavi et al., 2012). In fact, it is revealing that, of the 10,004 digital reconstructions currently in the online repository at Neuromorpho.org, only 24 are cerebellar neurons (as opposed to 5405 cerebral cortex neurons). In terms of digital reconstructions, the Purkinje neuron has been traced much more than other cerebellar neurons, perhaps because of its central role as the sole output neuron for the cerebellar cortex in tetrapods (Marr, 1969; Dean et al., 2010). The most complete Purkinje cell tracings are typically the result of injection techniques (e.g., Lucifer yellow: Sawada et al., 2010; biocytin: Roth and Häuser, 2001), and immunohistochemistry (Wu et al., 2010) with confocal laser microscropy, although the number of reconstructions usually remains small (<30). An even more limited number of Purkinje neuron reconstructions have been obtained using horseradish peroxidase and Golgi-Cox impregnations with light microscopy (Calvet and Calvet, 1984; Rapp et al., 1994; Milatovic et al., 2010). There appear to be no digital reconstructions of Purkinje neurons based on rapid Golgi stains. Finally, apart from a small number of traced molecular layer interneurons (*N* = 26; Sultan and Bower, 1998), there are few complete digital reconstructions of other neuronal types in cerebellar cortex.

In terms of comparative neuromorphology, research has generally focused on qualitative descriptions of Purkinje neurons. For example, there are well-documented morphological differences between tetrapods and teleosts such as the mormyrids, which have Purkinje neuron dendrites with a distinct palisade pattern (Meek and Nieuwenhuys, 1991). Quantitatively, however, there is very little comparative morphological information on cerebellar cortical neurons. To this end, the present study documents the morphological attributes of several types of cerebellar neurons. Following descriptions in rodents (Palay and Chan-Palay, 1974) and other mammals (rhesus monkey: Fox et al., 1967; cat: Larsell and Jansen, 1972; human: Braak and Braak, 1983), the superficial molecular layer contains the two-dimensional dendritic arrays of Purkinje neurons. These Purkinje neurons are described only qualitatively in the present study because rapid Golgi impregnations under light microscopy make complete and accurate tracings of their dense, distal dendritic segments extremely problematic, if not impossible. Also in the molecular layer are inhibitory interneurons, classically divided into (1) the relatively small stellate neurons in the outer two thirds of the layer, which are characterized by contorted, frequently dividing dendritic trees that radiate in multiple directions and by axons that are generally oriented horizontally; and (2) the somewhat deeper basket neurons, characterized by extensive, sea-fan shaped dendritic arbors and horizontally oriented axons that terminate in multiple pericellular baskets around the somata of Purkinje neurons. Although we follow the classical terminology for these interneurons in the present paper, it should be noted that both developmental research (Rakic, 1972) and empirical investigations (Sultan and Bower, 1998; Leto et al., 2006; Schilling et al., 2009) support early speculation (Ramón y Cajal, 1909, 1911) that these molecular inhibitory interneurons may actually be a uniform cell type whose ultimate morphology is determined by local cues at particular depths of the molecular layer.

Under the molecular layer, the Purkinje cell layer contains the large somata of Purkinje neurons, arranged in a single row, providing a clear demarcation between the other two layers. The deep granule cell layer contains the somata of two relatively large interneurons: (1) located immediately beneath the Purkinje cell layer, the Lugaro neurons (Golgi, 1874; Lugaro, 1894) are characterized by triangular or elongated fusiform shaped somata from which relatively long, thick, unbranched dendrites originate, typically extending in an arc under the Purkinje cell layer; and (2) somewhat deeper in the granule cell layer, the Golgi neurons (Golgi, 1874) are characterized by round somata with multiple dendrites radiating in all directions. Finally, throughout the granule cell layer are the very densely packed granule neurons, characterized by small, round somata extending several short, relatively unbranched dendrites characterized by gnarled, claw-like terminations.

The goals of the present comparative study were three-fold: (1) provide a qualitative description of neuronal morphology in the cerebellar cortex across the eight species examined; (2) provide quantitative data on the dendritic characteristics of these neurons; and (3) examine potential species differences in the dendritic measures of the traced neurons.

## Materials and methods

### Specimens

Tissue was obtained from eight species in the following phylogenetic groups: afrotherians (African elephant, Florida manatee), carnivores (Siberian tiger, clouded leopard), cetartiodactyls (humpback whale, giraffe), and primates (human, common chimpanzee). For captive animals (Siberian tiger, clouded leopard, chimpanzee), observations prior to death revealed no obvious behavioral abnormalities or deficits. Similar observations were not possible for animals in their natural habitat (African elephant, giraffe, humpback whale, Florida manatee, human). In post-mortem examinations, the brains of all animals exhibited no obvious abnormalities in terms of gross neuroanatomy. For five species (African elephant, Siberian tiger, clouded leopard, humpback whale, giraffe), cerebellar volume for at least one of the animals was obtained through magnetic resonance imaging (Maseko et al., 2012b). For the other species (Florida manatee, human, chimpanzee), direct measurement was not obtained because we did not have the opportunity for MRI scanning, nor was destructive dissection of the cerebellum an option. Instead, we had to rely on species mean values from the published literature. The present study was approved by the Colorado College Institutional Review Board (#011311-1) and the University of the Witwatersrand Animal Ethics Committee (2008/36/1).

#### African elephant (Loxodonta africana)

Cerebellar tissue from two 20 to 30-year-old, solitary male African elephants scheduled for population management culling was obtained after they were euthanized as described in Manger et al. (2009). *In situ* perfusion-fixation of the brains was conducted by removal of the head, flushing of the head with cold saline, and intra-carotid perfusion with 4% paraformaldehyde in 0.1 M phosphate buffer (autolysis time, AT, averaged = 135 min). The brains were then removed from the skull, placed in the same cold fixative and stored in 4% paraformaldehyde in 0.1 M phosphate buffer for 72 h. One brain had a mass of 5145 g and a cerebellar volume of 946 ml; the other brain had a mass of 4835 g and a cerebellar volume of 902 ml (Maseko et al., 2012b). Small tissue blocks containing the cerebellar regions of interest were stored in 0.1% sodium azide in 0.1 M phosphate buffer saline at 4°C for 8 months before Golgi staining.

#### Florida manatee (Trichechus manatus latirostris)

Following a watercraft collision in Florida, a sub-adult female manatee was euthanized. The head was perfused (by Roger L. Reep) via bilateral cannulation of the internal carotids, with 20 l phosphate buffer followed by 10 l of 4% paraformaldehyde. The brain (brain mass = 316 g; estimated cerebellar volume = 44 ml; Reep and O'Shea, 1990) was removed (AT = 6 h) and stored in a cold 2% paraformadehyde solution for ~2 days. One cerebellar tissue block was removed and stored in cold (2°C) phosphate buffer solution for 3 additional days before Golgi staining.

#### Siberian tiger (Panthera tigris altaica)

One 12-year-old female from the Copenhagen Zoo in Denmark was euthanized. *In situ* perfusion-fixation (by Mads F. Bertelsen) of the brain (AT < 30 min) followed the same protocol as in the elephant (brain mass = 258 g; cerebellar volume = 37 ml). Cerebellar tissue blocks were stored in 0.01% sodium azide in 0.1 M phosphate buffer saline at 4°C for 6 months before Golgi staining.

#### Clouded leopard (Neofelis nebulosa)

Two adult female clouded leopards were euthanized for medical reasons: a 20-year old from the Smithsonian National Zoological Park in Washington, DC., and a 28-year old from the Cleveland Metroparks Zoo (AT < 30 min for both animals). The brains were immersion fixed in 10% formalin for 10 (20-year old) and 34 days (28-year old). Brain mass was 82 g for the 20-year old and 73 g for the 28-year old; cerebellar volume was an average of 8.6 ml for both animals. Subsequently, the brains were stored in 0.1% sodium azide in 0.1 M phosphate buffer saline at 4°C prior to Golgi staining (5 months for the 20-year old; 3 years for the 28-year old).

#### Humpback whale (Megaptera novaeangliae)

A ~2 year-old male humpback whale, 9.45 m in length, was stranded in East Hampton, Long Island, New York in April, 2010. A necropsy was performed (by Patrick R. Hof, Bridget Wicinski, and Camilla Butti) immediately after death. The brain (brain mass = 3606 g; cerebellar volume = 695 ml) was removed (AT = 8 h) and immersion-fixed in 4% paraformaldehyde for 2 years prior to Golgi staining.

#### Giraffe (Giraffa camelopardalis)

The brains of three solitary, free ranging, sub-adult (~2–3 years of age) male giraffes were obtained and processed in the same manner as the elephant (Dell et al., 2012). Brain masses—cerebellar volumes for these three animals were 610 g—83 ml, 527 g—69 ml, and 480 g—67 ml. Cerebellar blocks were stored in 0.1% sodium azide in 0.1 M phosphate buffer for 4 months prior to Golgi staining.

#### Human (Homo sapiens)

Human tissue was provided by Dr. R. Bux of the El Paso County coroner's office in Colorado Springs. Tissue blocks were removed from the cerebellum of a neurologically normal, 54-year-old male who had died of acute myocardial infarction (brain mass = 1435 g; estimated cerebellar volume = 139 ml; Smaers et al., 2011; Maseko et al., 2012b). Tissue was immersion fixed in 10% formalin and stored at 2°C for ~1 week before Golgi staining (AT = 5 h).

#### Common chimpanzee (Pan troglodytes)

Two adult chimpanzees were obtained from the Yerkes National Primate Center: a 23-year-old female who died under anesthesia, and a 39-year-old male euthanized due to congestive heart failure. Brains were immersion fixed in 10% formalin (13 days for the 23-year old; 4 months for the 39 year old; AT < 1 h). Subsequently, brains were stored in 0.1% sodium azide in 0.1 M phosphate buffer saline at 4°C prior to Golgi staining (4 years for the 23-year old; 2 years for the 39-year old). Brain mass was 408 g for the 23-year old and 392 g for the 39-year old; cerebellar volume was estimated to be an average of 43 ml for both animals (Smaers et al., 2011; Maseko et al., 2012b).

### Tissue selection

**Figure 1:**
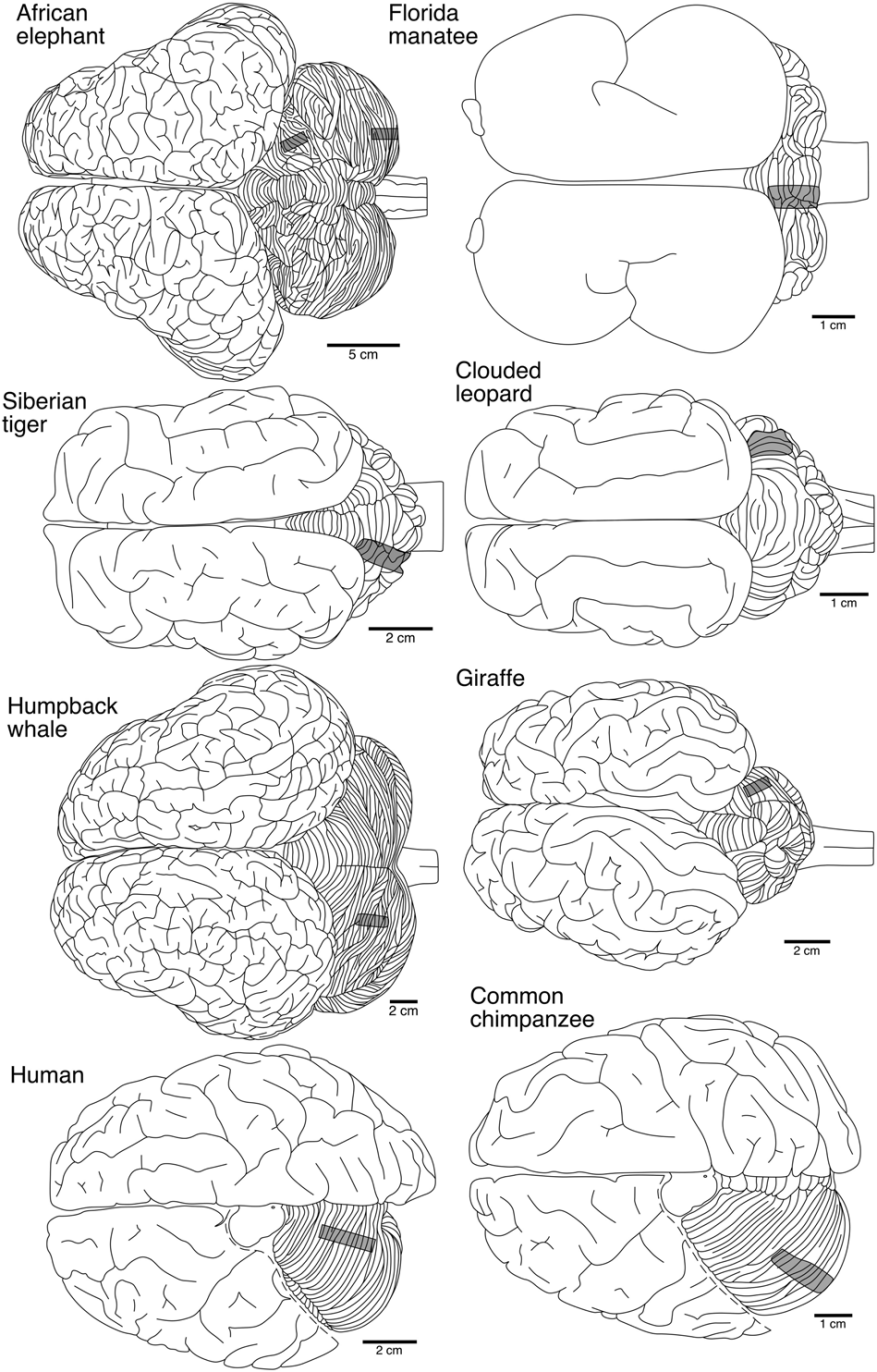
**Dorsal views of the brains from the eight species in the current study illustrating the relative location from which tissue blocks were selected from the cerebellum for staining**. Represented from top to bottom are: afrotherians (African elephant, Florida manatee), carnivores (Siberian tiger, clouded leopard), cetartiodactyls (humpback whale, giraffe), and primates (human, common chimpanzee). For the two primates, the dorsal portion of the cerebrum has been removed to reveal the cerebellum. Note scale bar is different for each species.

In five of the species (Florida manatee, Siberian tiger, humpback whale, human, and chimpanzee), tissue blocks (3–5 mm thick) were removed from the dorsal posterior aspect of the posterior lobe and from the dorsal anterior aspect of the anterior lobe of the left cerebellar hemisphere (Figure 1). In the remaining three species (African elephant, giraffe, and clouded leopard), the same regions were sampled from the right cerebellar hemisphere. Tissue was coded to prevent experimenter bias, stained via a modified rapid Golgi technique (Scheibel and Scheibel, 1978), and sectioned serially perpendicular to the long axis of the folia at 120 μm with a vibratome (Leica VT1000S, Leica Microsystems, Inc.). Because of the small number of neurons traced in each species, neurons from anterior and posterior cerebellar lobes were combined for all subsequent analyses.

### Neuron selection and quantification

Neurons were selected for tracing based on established criteria (Roitman et al., 2002; Anderson et al., 2009; Jacobs et al., 2011; Lu et al., 2013), which required an isolated, darkly stained soma near the center of the 120 μm section, with as fully impregnated, unobscured, and complete dendritic arbors as possible (i.e., no beading or interruptions). In the tracing process, dendritic branches were not followed into adjacent sections. Although serial section reconstructions of dendrites are possible with some histological techniques (e.g., intracellular injections), accurate reconstructions are problematic in Golgi stained material, where multiple neural elements overlap in the same section. As such, only portions captured in the 120 μm-thick section could be compared in the present study, resulting in an overall underestimation of dendritic values insofar as neurons with longer dendrites are disproportionately cut in the sectioning process. Prior to quantification, Golgi-stained sections were examined to determine neuronal types. The neurons of interest included the molecular layer interneurons (e.g., stellate, basket), as well as Lugaro, Golgi, and granule cells. As noted above, we kept the classical distinction between stellate and basket neurons for the molecular layer interneurons, although it has been argued that they actually constitute the same neuronal population (Sultan and Bower, 1998). Additionally, no distinction was made between superficial and deeper stellate neurons, or between large and small Golgi neurons (Palay and Chan-Palay, 1974). Purkinje neurons were photomicrographed but not traced due to the complexity of their dendritic plexus. Candelabrum (Lainé and Axelrad, 1994), unipolar brush (Altman and Bayer, 1977), and synarmotic (Landau, 1933; Flace et al., 2004) neurons were not observed in the current preparations. Certain morphological characteristics of neurons traced in the elephant have previously been reported (Maseko et al., 2012a), but are included here with a more in-depth analysis. Golgi impregnation was inconsistent across species, both in terms of overall numbers of neurons and the types of neurons that stained. Consequently, neurons traced in different individuals within a species (i.e., elephant, clouded leopard, giraffe, chimpanzee) were combined without consideration of individual differences.

Quantification was performed under a Planachromatic 60x oil objective (N.A. 1.4), except for elephant neurons, which were quantified under a Planachromat 40x dry objective (N.A. 0.70). Based on prior research, this difference in microscope objectives was not expected to significantly affect dendritic measures (Anderson et al., 2010). Neurons were traced along x-, y-, z-coordinates using a Neurolucida system (MBF Bioscience, Williston, VT) interfaced with an Olympus BH-2 microscope equipped with a Ludl XY motorized stage (Ludl Electronics, Hawthorne, NY) and a Heidenhain z-axis encoder (Schaumburg, IL). A MicroFire Digital CCD 2-megapixel camera (Optronics, Goleta, CA) mounted on a trinocular head (model 1-L0229, Olympus, Center Valley, PA) displayed images on a 1920 × 1200 resolution Dell E248WFP 24-inch LCD monitor. Somata were traced first at their widest point in the 2-dimensional plane to provide an estimate of the cross-sectional soma area, a measure that appears highly correlated with soma volume (Ulfhake, 1984). Subsequently, dendrites were traced somatofugally in their entirety, recording dendritic diameter. Dendritic arbors with unclear, sectioned, broken, ambiguous, or obscured terminations were identified as incomplete endings. Of the 13,698 dendritic segments quantified, 45% were intermediate segments. With regard to terminal segments, 58% were complete, and 42% were incomplete, which is a higher completion ratio than obtained in neocortex with the same methodology (Jacobs et al., 1997, 2001). Neurons with sectioned segments were not differentially analyzed because elimination of neurons with incomplete segments would have biased the sample toward smaller neurons (Schadé and Caveness, 1968; Uylings et al., 1986).

Neurons were traced by three investigators (Busisiwe C. Maseko, Nicholas L. Johnson, Devin Wahl). Intrarater reliability was determined by having each rater trace the same soma and dendritic segment 10 times. The average coefficient of variation for soma size (2.5%) and total dendritic length (TDL, 2.8%) indicated little variation in the tracings. Intrarater reliability was further tested with a split plot design (α = 0.05), which indicated no significant difference between the first five tracings and the last five tracings. Interrater reliability was determined through comparison of 10 dendritic system tracings with the same tracings completed by the primary investigator (Bob Jacobs). Interclass correlations across soma size and TDL averaged 0.99 and 0.99, respectively. An analysis of variance (ANOVA; α = 0.05) failed to indicate significant differences among the tracers for the three measures. Additionally, the primary investigator reexamined all completed tracings under the microscope to ensure accuracy.

### Cell descriptions and dependent dendritic measures

Neurons were classified according to somatodendritic morphological characteristics, closely following well-established descriptive criteria (Ramón y Cajal, 1909, 1911; Rakic, 1972; Palay and Chan-Palay, 1974; Braak and Braak, 1983; Melik-Musyan and Fanardzhyan, 2004). Quantitatively, soma size (i.e., surface area, μm^2^) and depth from the pial surface (μm) were measured. Dendritic branches extending from the soma were characterized centrifugally (Bok, 1959; Uylings et al., 1986), and quantified with four previously established measures (Jacobs et al., 2011): dendritic volume (Vol, μm^3^; the total volume of all dendrites); total dendritic length (TDL, μm; the summed length of all dendritic segments); mean segment length (MSL, μm; the average length of each dendritic segment); and dendritic segment count (DSC; the number of dendritic segments). One additional measure from Maseko et al. (2012a) was examined: dendritic tortuosity (Tor), a measure of the relative straightness-twistedness of a dendrite. The tortuosity index was calculated by dividing the total length of dendritic segments from the origin point on the soma to the end point by the length of a vector: an index of 1 equals a straight line; an index greater than 1 means the path of the dendrites is more complex than a straight line (Foster and Peterson, 1986; Wen et al., 2009). Finally, dendritic branching patterns were analyzed using a Sholl analysis (Sholl, 1953), which quantified dendritic intersections at 20-μm intervals radiating somatofugally.

### Inferential statistical analyses of interspecies differences

Measures (Vol, TDL, MSL, DSC, Tor) for every dendritic segment, along with soma size and depth, were aggregated for each neuron (SPSS release 20.0.0). A total of 317 neurons were traced in 12 members of 8 species, with the following breakdown: African elephant (*n* = 20; 18 in one animal, 2 in the other), Florida manatee (*n* = 25), Siberian tiger (*n* = 33), clouded leopard (*n* = 32; 21 in the 20-year old; 11 in the 28-year old), humpback whale (*n* = 47), giraffe (*n* = 56; 21, 21, and 14 in each of the three animals), chimpanzee (*n* = 86; 51 in the 23-year old; 35 in the 39-year old), human (*n* = 28). As indicated in Table 1, neuron types were unevenly distributed among species and some neuron types did not stain in some species (i.e., granule neurons in afrotherians, and Golgi neurons in the manatee).

**Table 1:** **Number of tracings for each neuronal type within each species**.

**Species**	**Neuron type**					
	**Stellate**	**Basket**	**Lugaro**	**Golgi**	**Granule**	**Total**
African elephant	5	5	5	5	0	20
Florida manatee	6	8	1	0	0	15
Siberian tiger	5	8	3	7	10	33
Clouded leopard	16	5	4	1	6	32
Humpback whale	9	11	11	6	10	47
Giraffe	17	13	4	7	15	56
Human	7	6	5	5	5	28
Common chimpanzee	20	16	17	17	16	86
Total	85	72	50	48	62	317

For inferential analyses, we initially constructed a table of dendritic measures for all neuron types to explore differences among species (Table 2). Given that cerebellar volume increases with the size of the brain (Smaers et al., 2011; Maseko et al., 2012b), the table was used to evaluate if the same is true for dendritic measures. Confidence intervals of 95% were constructed for each measure. Because elephants had the largest cerebellar volume (averaging 924 ml) and clouded leopards the smallest (averaging 8.6 ml), these two species were used as the references against which the confidence intervals for the other species were compared. Unfortunately, because only one Golgi neuron stained in the clouded leopard, we could not construct confidence intervals for that species and neuron; instead, only the elephant was used as a reference for Golgi neurons. Similarly, the elephant had no stained granule neurons, so only the clouded leopard was used for comparison. In examining the table columns for each type of dendritic measure, any species-specific value outside the reference indicates a difference between the two distributions. The goal was to evaluate how much, or how little, the reference distributions for elephant and clouded leopard overlapped with the distributions of the dendritic values for the other species. By comparing the amount of overlap between the distributions, a much better idea of the comparative distributions was provided than if just *F*-tests based on means had been presented. Significant differences were highlighted in bold for comparison to the elephant and red for comparisons with the clouded leopard. Because of the usage of 95% confidence intervals, any two distributions that did not overlap were different at *p* ≤ 0.05. For example, in Table 2, the TDL lower (176.5) and upper confidence intervals (2735.2) for Golgi neurons in the chimpanzee are in bold text, indicating that the Golgi TDL values in the chimpanzee (1455.9 ± 639.7 μm) are significantly [*F*_(1315)_ = 720.62, *p* ≤ 0.05] different from those in the reference animal, namely the elephant (Golgi TDL = 5664.2 ± 878.9 μm). Although Table 2 provides detailed information, it does not specify whether any of the species can be identified from just dendritic characteristics. Because accurate differentiation of species may require combinations of multiple dendritic measures, a more comprehensive analysis was necessary.

**Table 2:**
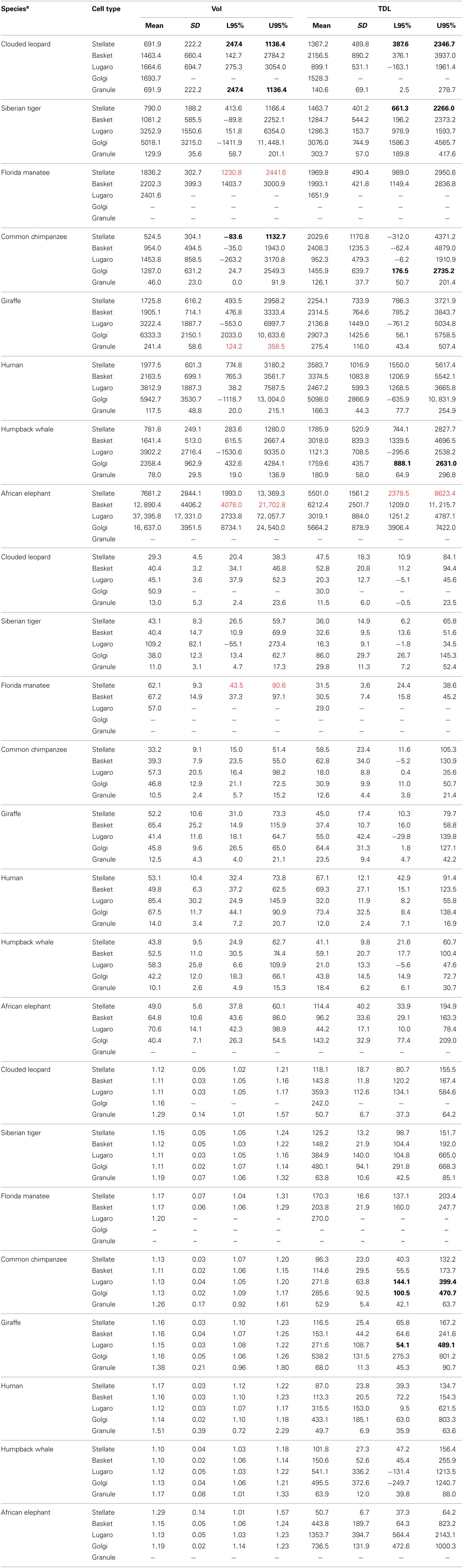
**Dendritic measures and soma size for each neuronal type across species^a^**.

*^a^Dependent measures are: Volume (Vol, μm^3^), Total Dendritic Length (TDL, μm), Mean Segment Length (MSL), Dendritic Segment Count (DSC), Tortuosity (Tor), and Soma Size (μm^2^). Species are arranged from smallest (clouded leopard) to largest (African elephant) in terms of brain mass. L95% = lower 95% confidence interval; U95% = upper 95% confidence interval. Bold numbers represent comparisons that were significantly different from the elephant values; red numbers represent comparisons that were significantly different from the clouded leopard values. See text for further explanation*.

There are six potential analytic obstacles to overcome in the present, or any, quantitative neuromorphological analysis. First, the brains for each species were fixed and preserved differently, which contributed to “noise,” or error variability in measurements both within the same species and between different species. Second, differential stain impregnation introduced measurement error at the dendritic level. Third, some neuron types did not stain in some animals. Fourth, the standard assumption of any conventional statistical analysis that requires the use of covariances (e.g., principal components, ordinary least squares regression, and even Pearson correlations) is that error terms are uncorrelated (Williams et al., 2013). This requirement is not met when, between variables, there are relationships that are due to multiple sources. Such is the case in neuroanatomy, where animals have both a genetic and a socio-developmental background. The confluence of these phylogenetic and ontogenetic factors shape the underlying neuronal morphology of an individual animal as well as the common characteristics of conspecifics. Consequently, it is not possible to evaluate the null hypothesis in the present study with a conventional statistical test (Williams et al., 2013). Fifth, sample size constrains the ability to use statistics to test differences between members of a species or between species themselves. One common rule for designs with nested levels within groups calls for a minimum of 30 units at each level of measurement (Bell et al., 2008). A study of dendritic characteristics between two species would require 30 specimens from each, with a sample of enough neurons to generate 30 randomly selected neurons, from which 30 randomly chosen dendritic trees would be selected, and so on. It is highly unlikely that a researcher would have access to 60 animals divided equally between two species, or the ability to stain and trace a minimum of 1800 complete dendritic trees. Sixth, inferential statistical methods such as *t*-tests and ANOVAs, with their standard errors, test statistics, and *p*-values require that study samples be randomly selected (Friedman, 1991; Berk and Freedman, 2003). In comparative neuromorphology research, there are no random samples where each unit analyzed has an equal probability of being selected. All samples in such studies are those of convenience, and may not represent any definable population larger than itself (Freedman, 2004).

The present study therefore sought to employ an analytic method that could provide solid evidence of the ability to differentiate species from dendritic measures despite the variability introduced by factors such as differential brain fixation and neuronal staining. Moreover, the analyses had to make these predictions despite violations of the uncorrelated error, random sample, and sample size requirements, which invalidate the use of any conventional statistical tests (e.g., *F*-tests or principal components). Thus, we were limited to nonparametric techniques that make no assumptions about distributional, correlational, random sampling, or other requirements. The technique chosen is referred to as MARS, MARSplines, or Multivariate Adaptive Regression Splines (Statistica, release 12; StatSoft Inc, Austin, TX; Friedman, 1991; Hastie et al., 2009). The MARSplines technique is appropriate because it does not assume or impose any restrictions or conditions for the differentiation of species with dendritic measures, and it can create useful models even in quite difficult situations similar to those faced in quantitative neuromorphology (http://sdn.statsoft.com/STATISTICAVisualBasic.aspx?page=category&item=modules%3AStatistics%3ASTAMARSplines). Further, examination of the mathematics of MARSplines demonstrates that there are no distributional assumptions or sample size requirements for the *r*^2^ statistic it generates. For these reasons, the MARSplines analysis was employed in the present study to explore potential species differences in dendritic measures.

## Results

### Overview

In terms of gross anatomy across the sampled species, there was a 64-fold variation in brain mass (from an average of 78 g in the leopards to an average of 4990 g in the elephants) and a 103-fold variation in cerebellar volume (from an average of 9 ml in the leopards and an average of 924 ml in the elephants). The larger variation in cerebellar volume appears to be a result of a disproportionately large cerebellum in the elephant (Maseko et al., 2012b). A Spearman's rho correlation between brain mass and cerebellar volume revealed a strong positive relationship [*r*_(13)_ = 0.977, *p* = 0.01]. Cerebellar volume averaged 13.6 ± 3.1% of total brain mass, with the following breakdown: whale (18.6%), elephant (18.5%), giraffe (13.6%), tiger (13.8%), manatee (13.4%), leopard (11.1%), chimpanzee (10.4%), and human (9.3%). Dendritic measures and soma size also tended to increase with cerebellar volume for most neuronal types, particularly in terms of dendritic Vol and TDL (Table 3). However, removing the elephant data resulted in a 32% decrease in the magnitude of these correlations and a reduction in the number of significant correlations from 17 to 8 (Table 3), suggesting that the elephant measures were skewing overall results.

**Table 3:**
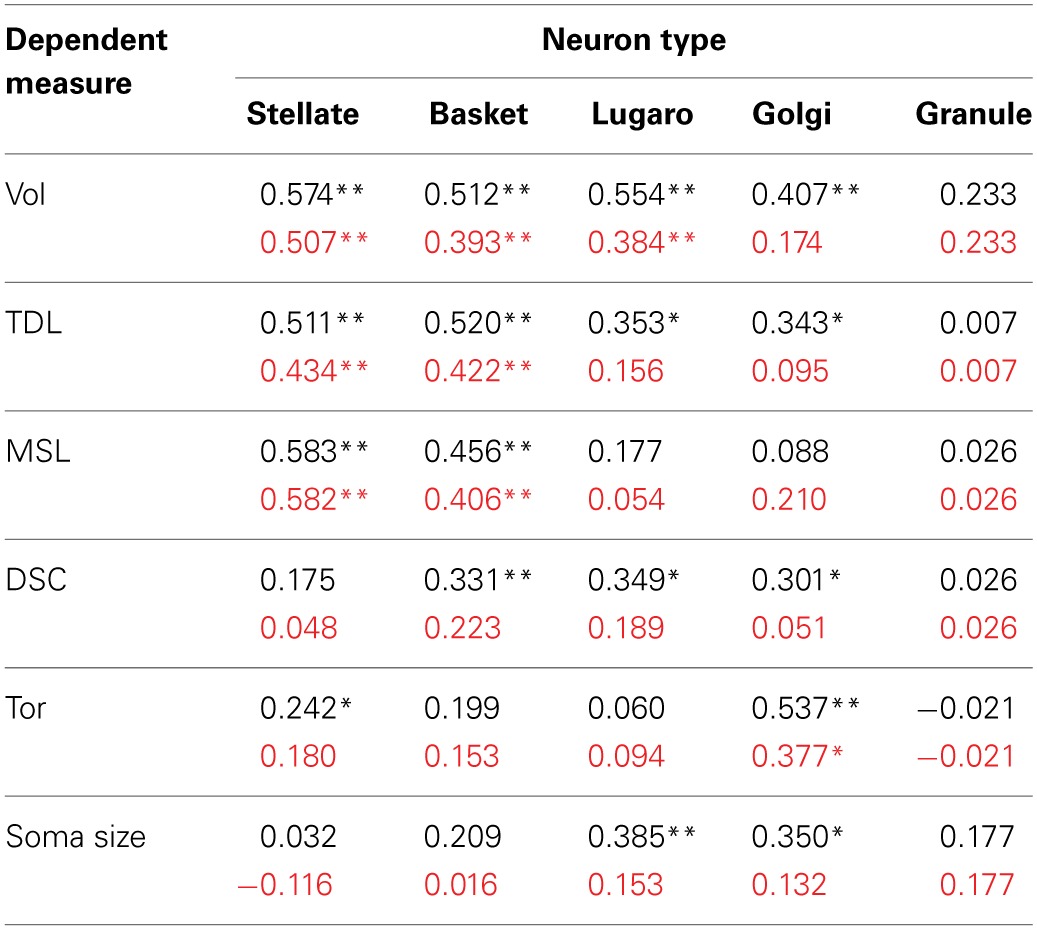
**Matrix of Spearman's rho correlations between cerebellar volume and dendritic measures (and soma size) across all neuron types^a^**.

^a^Correlations in black font are for all eight species (N = 317 neurons); correlations in red font are for all species except the elephant (N = 297 neurons). Note the reduction in the magnitude of all correlations when the elephant is removed from the analysis. ^*^p = 0.05; ^**^p = 0.01.

**Figure 2:**
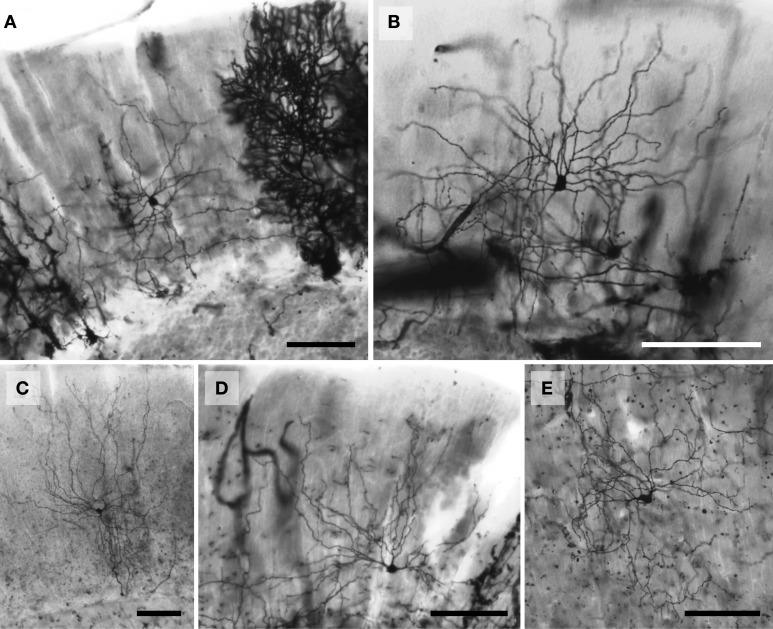
**Photomicrographs of Golgi-stained stellate neurons. (A)** Florida manatee; **(B)** chimpanzee (see also Figure 11Q); **(C)** human (see also Figure 11D); **(D)** giraffe; and **(E)** African elephant (see also Figure 8C). Scale bars: 100 μm.

**Figure 3:**
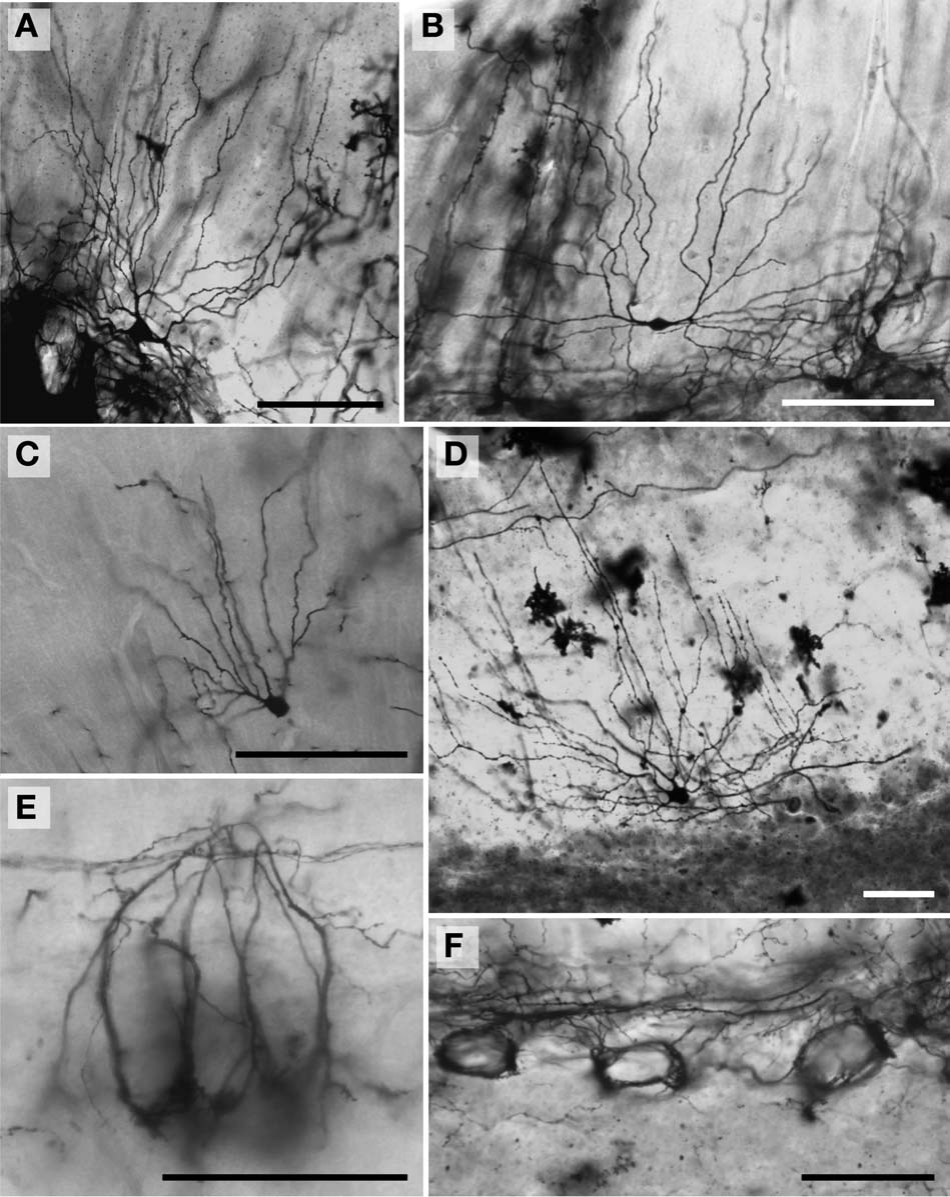
**Photomicrographs of Golgi-stained basket neurons. (A)** Humpback whale; **(B)** chimpanzee; **(C)** giraffe; and **(D)** African elephant. The pericellular baskets encapsulating Purkinje cell bodies (unstained) are represented in **(E)** Siberian tiger and **(F)** humpback whale. Scale bars: 100 μm.

**Figure 4:**
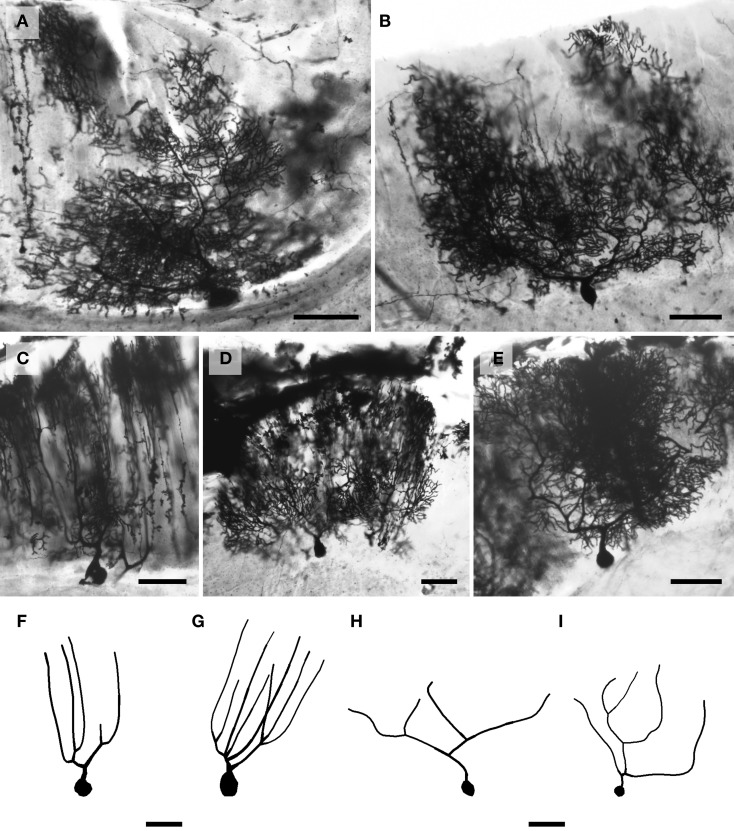
**Photomicrographs of Golgi-stained Purkinje neurons. (A)** African elephant; **(B)** human; **(C)** humpback whale; **(D)** Florida manatee; and **(E)** giraffe. Note the large similarity between the neurons of all species except the humpback whale, where the Purkinje neuron appears to have more vertically oriented tertiary branches. Sample tracings of the main dendritic branches illustrating this morphological difference are provided for the humpback whale **(F,G)**, which differs substantially even from the other cetartiodactyl in the study, the giraffe **(H,I)**. Scale bars: 100 μm.

**Figure 5:**
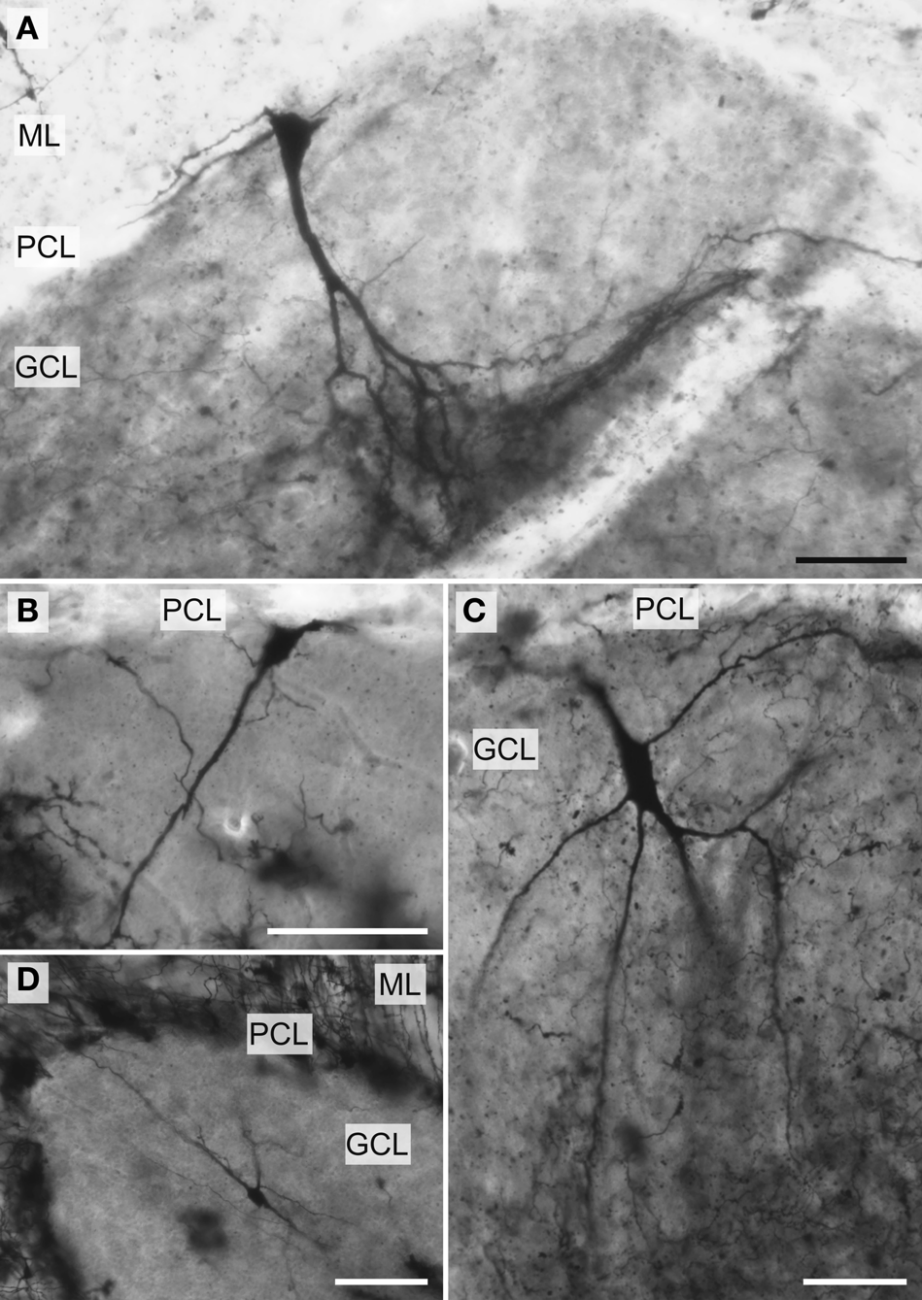
**Photomicrographs of Golgi-stained Lugaro neurons**. (**A**, see also Figure 8J) and (**C**, see also Figure 8L) African elephant; in these two neurons, note the bouquet shaped dendritic arbor in **(A)** and the more solitary, unbranched dendritic arbor in **(C)**, with both descending to the underlying white matter. The **(B)** humpback whale (see also Figure 10L) and **(D)** chimpanzee also have predominantly unbranched dendritic trees. ML, molecular layer; PCL, Purkinje cell layer; GCL, granule cell layer. Scale bars: 100 μm.

**Figure 6:**
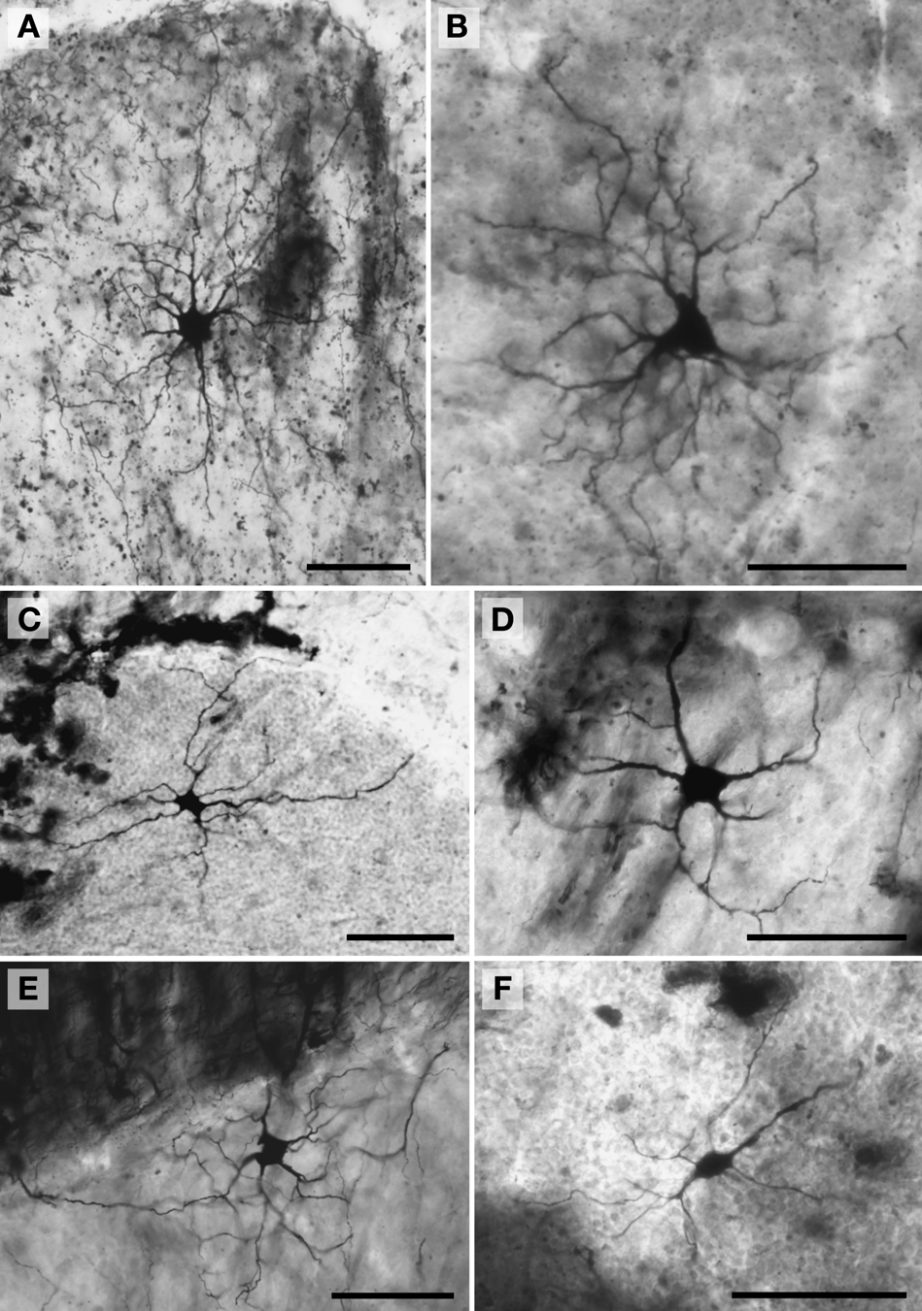
**Photomicrographs of Golgi-stained Golgi neurons. (A)** and (**B**, see also Figure 8H) African elephant; **(C)** chimpanzee (see also Figure 11GG); **(D)** Siberian tiger (see also Figure 9E); **(E)** giraffe (see also Figure 10DD); and **(F)** clouded leopard (see also Figure 9BB). Scale bars: 100 μm.

**Figure 7:**
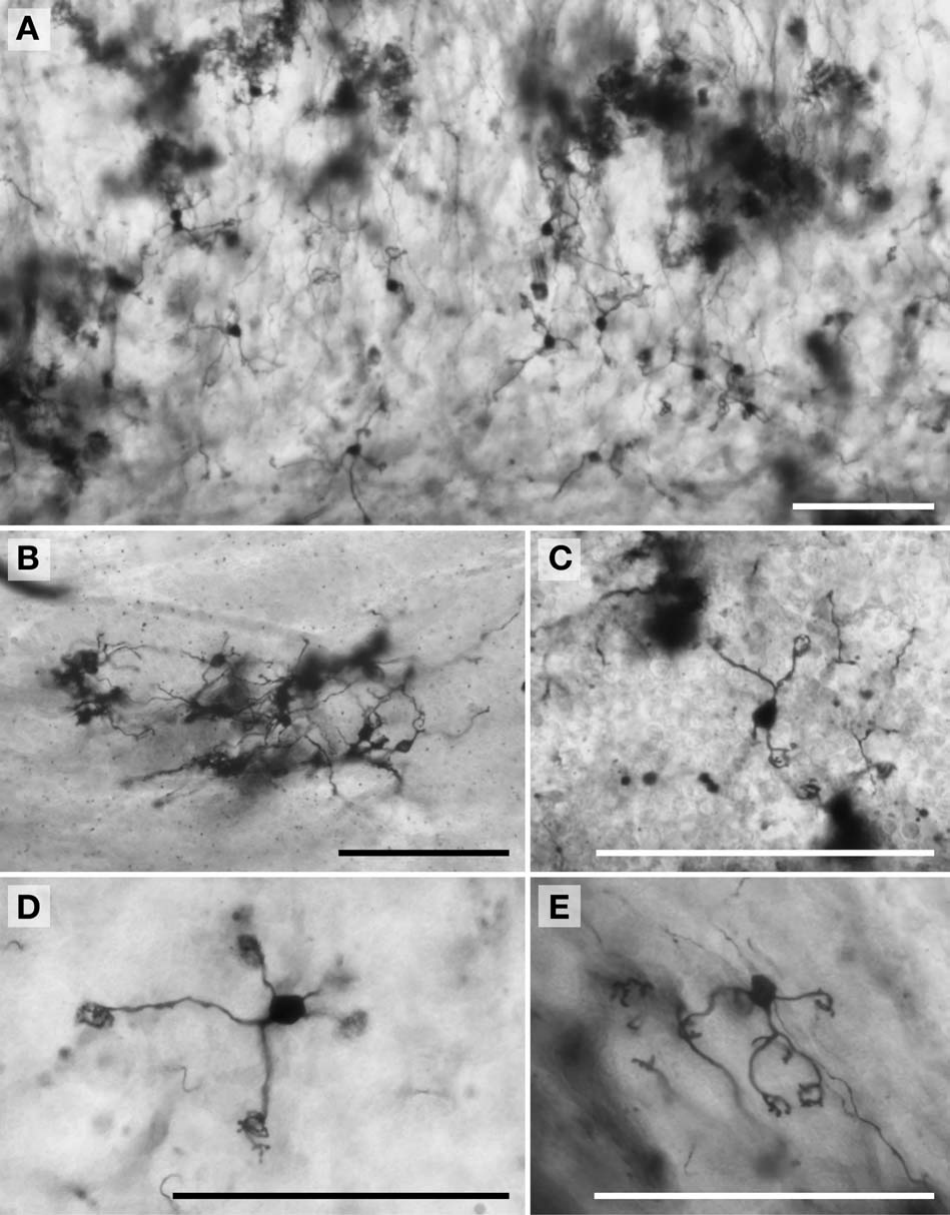
**Photomicrographs of Golgi-stained granule neurons. (A)** Siberian tiger; **(B)** humpback whale; **(C)** chimpanzee; **(D)** Siberian tiger; and **(E)** giraffe. Scale bars: 100 μm.

Histologically, there was considerable variation in the Golgi stain across species. Each impregnation was nevertheless of sufficient quality to allow for adequate quantification of selected neurons (Figures 2–7). The expected trilaminate architecture of cerebellar cortex was present in all species. The molecular layer, similar to supragranular layers in the cerebral neocortex (Jacobs et al., 1997, 2001), tended to stain better (i.e., exhibited a clearer background with less obstructed, more complete neurons) than the deeper granule cell layer, which allowed more molecular than granular layer inhibitory interneurons to be traced (Table 1). A Spearman's rho correlation indicated a significant (*p* = 0.01) positive relationship between soma size and all dendritic measures in the total sample [Vol: *r*_(317)_ = 0.826; TDL: *r*_(317)_ = 0.497; MSL: *r*_(317)_ = 0.647; DSC: *r*_(317)_ = 0.253] except Tor, which was negative [*r*_(317)_ = −0.285]. In terms of morphology, traced neurons tended to be similar across all species. When comparing across all neuron types, molecular layer interneurons consistently fell in the middle of all dendritic measures. The largest neurons traced were the Lugaro neurons (dendritic Vol ranged from 1454 μm^3^ in the chimpanzee to 37,396 μm^3^ in the elephant) and the Golgi neurons (TDL ranged from 1456 μm in the chimpanzee to 5664 μm in the elephant). Lugaro neurons also tended to have the longest MSL values (ranging from 41 μm in the giraffe to 109 μm in the tiger) whereas Golgi neurons tended to have the highest DSC values (ranging from 30 in the leopard to 143 in the elephant). Granule neurons exhibited the lowest values for every measure except Tor, which obtained its highest value in granule neurons (ranging from 1.17 in the humpback whale to 1.51 in the human).

**Figure 8:**
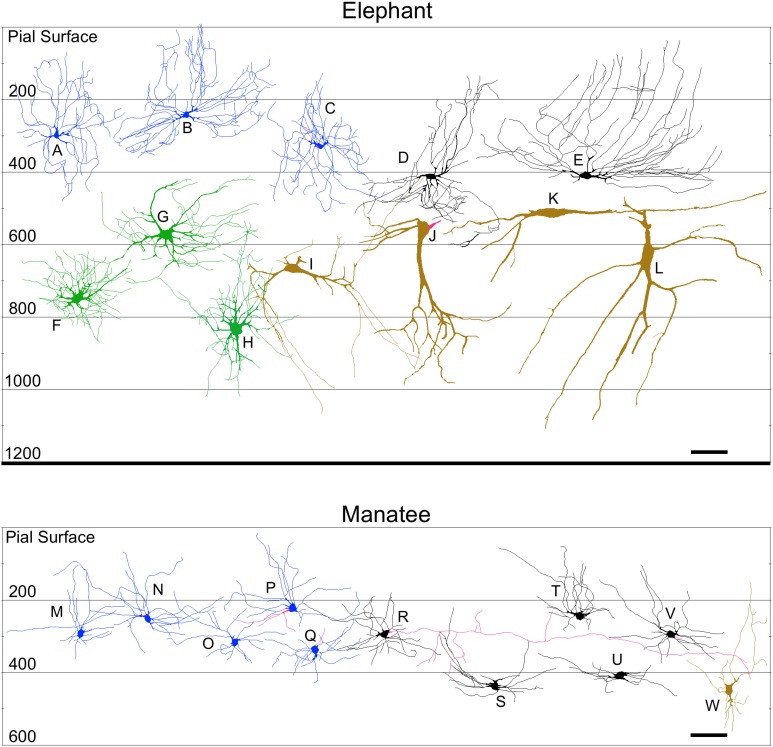
**Neurolucida tracings of neurons in the cerebellar cortex of the African elephant (top) and Florida manatee (bottom) indicating relative soma depth from the pial surface (in μm)**. Stellate neurons **(A–C**; **M–Q)**; basket neurons **(D,E**; **R–V)**; Lugaro neurons **(I–L; W)**; Golgi neurons **(F–H)**. Scale bar: 100 μm.

**Figure 9:**
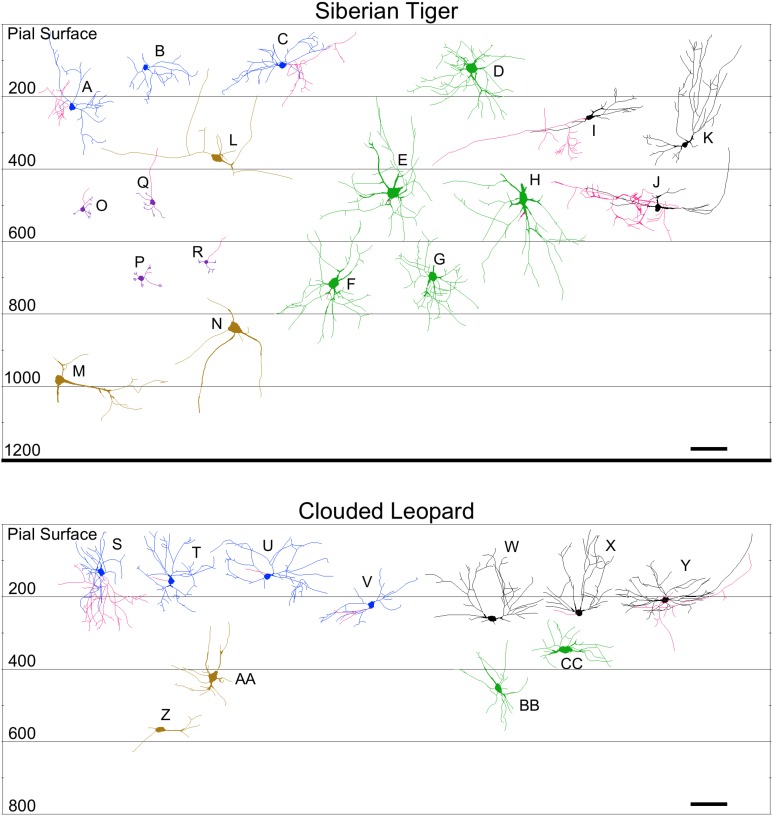
**Neurolucida tracings of neurons in the cerebellar cortex of the Siberian tiger (top) and clouded leopard (bottom) indicating relative soma depth from the pial surface (in μm)**. Stellate neurons **(A–C; S–V)**; basket neurons **(I–K**; **W–Y)**; Lugaro neurons **(L–N**; **Z–AA)**; Golgi neurons **(D–H**; **BB,CC)**; granule neurons **(O–R)**. Axons, when present, are indicated in red. Scale bar: 100 μm.

**Figure 10:**
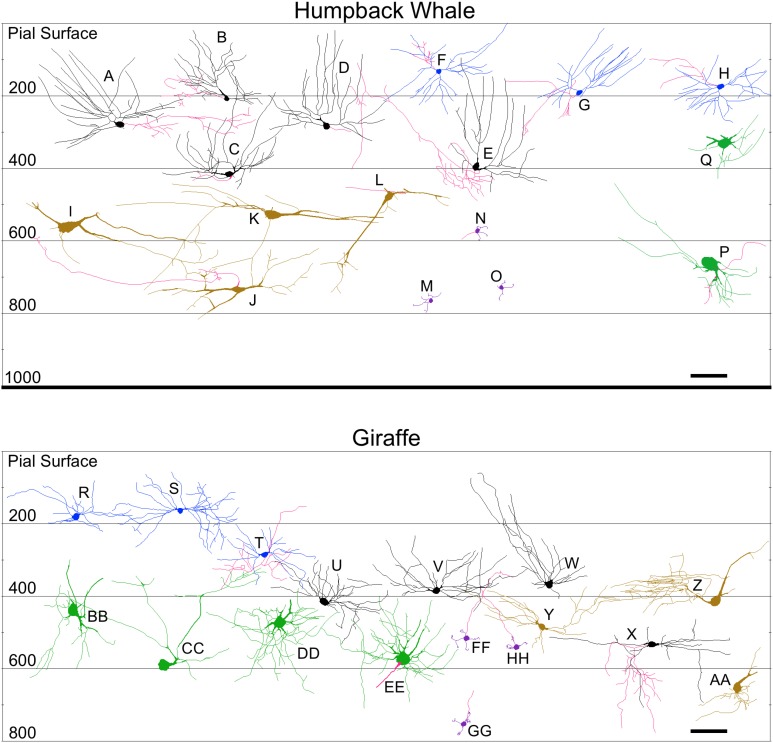
**Neurolucida tracings of neurons in the cerebellar cortex of the humpback whale (top) and giraffe (bottom) indicating relative soma depth from the pial surface (in μm)**. Basket neurons **(A–E**; **U–X)**; stellate neurons **(F–H; R–T)**; Lugaro neurons **(I–L; Y–AA)**; Golgi neurons **(P,Q; BB–EE)**; granule neurons **(M–O; FF–HH)**. Axons, when present, are indicated in red. Scale bar: 100 μm.

**Figure 11:**
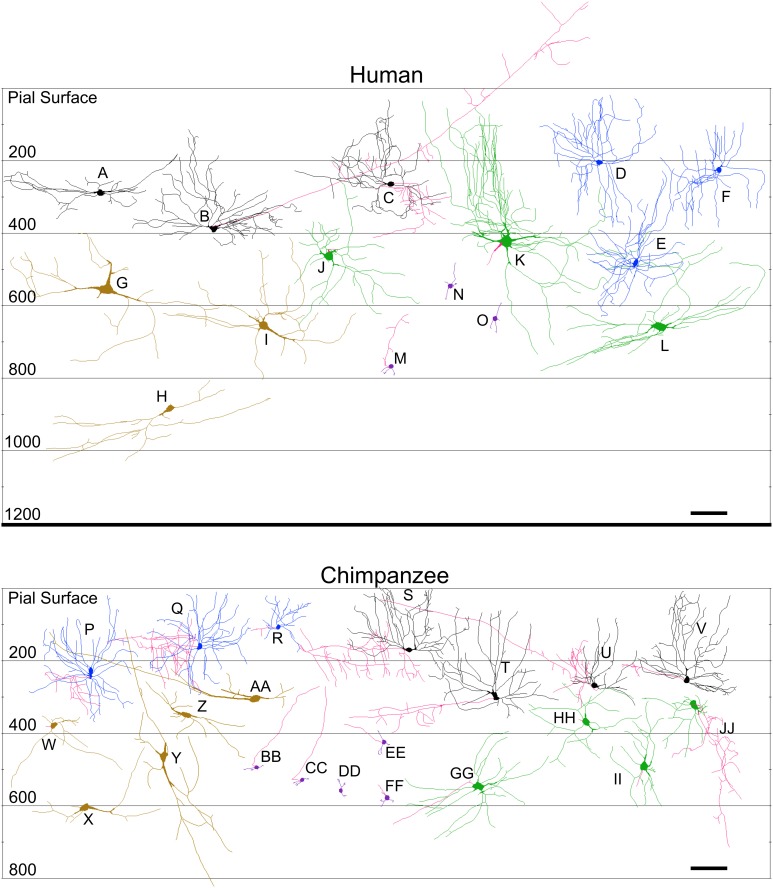
**Neurolucida tracings of neurons in the cerebellar cortex of the human (top) and chimpanzee (bottom) indicating relative soma depth from the pial surface (in μm)**. Basket neurons **(A–C; S–V)**; stellate neurons **(D–F; P–R)**; Lugaro neurons **(G–I; W–AA)**; Golgi neurons **(J–L**; **GG–JJ)**; granule neurons **(M–O**; **BB–FF)**. Axons, when present, are indicated in red. Note that the axons for basket neurons **(B)** and **(U)** followed the curvature of the folia in their original sections for a long distance, and thus incorrectly appear, here in the schematic, to extend to or beyond the pial surface. Scale bar: 100 μm.

**Figure 12:**
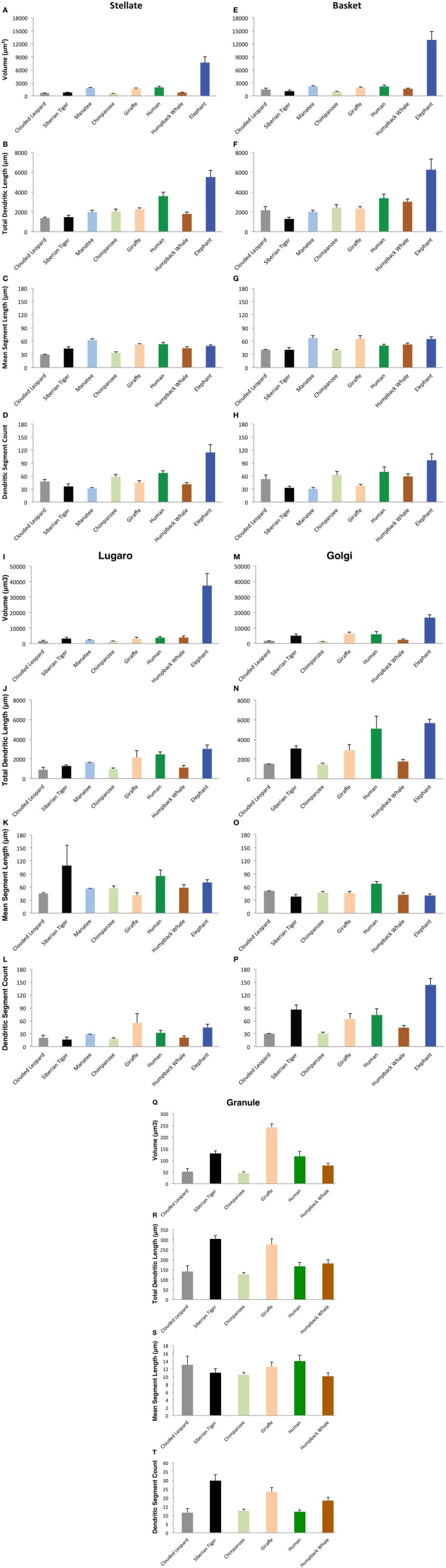
**Bar graphs indicated the relative values of four dependent measures (Volume, Total Dendritic Length, Mean Segment Length, and Dendritic Segment Count) for stellate **(A–D)**, basket **(E–H)**, Lugaro **(I–L)**, Golgi **(M–P)**, and granule **(Q–T)** neurons across the eight species in the current study**. The eight species are arranged from left to right on the abscissa in a fixed order, from the smallest (clouded leopard) to the largest brain (elephant). Phylogenetic relationships among species are color coded as follows: afrotherians (dark blue = elephant; light blue = manatee); carnivores (black = tiger; gray = leopard); cetartiodactyls (dark brown = humpback; light brown = giraffe); and primates (dark green = human; light green = chimpanzee). Note the following: (1) tortuosity measures are not illustrated here; (2) granule neurons in afrotherians, and Golgi neurons in the manatee are not illustrated here because they did not stain; and (3) the ordinate scale for granule neurons is much smaller than the scale for other neuron types. Error bars = s.e.m.

Sample tracings of neuronal types for each species are provided in Figures 8–11. Mean values of selected dependent measures (i.e., Vol, TDL, MSL, DSC) for each neuronal type across species are presented in Figure 12. Although the graphs in Figure 12 illustrate mean values, only the ranges across species are used in the text below for these dendritic measures because (1) there is asymmetric variation in the dependent measures across and within species and neuronal types, and (2) the extremely large values exhibited by the elephant for most of the dendritic measures distort the overall means. The morphological characteristics of these neuronal types are addressed in detail below, followed by the results from interspecies comparisons of dendritic measures.

### Molecular layer

*Stellate neurons* (Figure 2) were the most superficial neurons traced (*Mean*_soma depth_ = 2.3 ± 108 μm). Their round or ovoid somata were smaller than all other neurons except granule cells, with a 2.13-fold range in size across species (chimpanzee = 86 μm^2^ < human < whale < giraffe < leopard < tiger < manatee < elephant = 183 μm^2^). Sample tracings of stellate neurons are provided for each species: African elephant (Figures 8A–C), Florida manatee (Figures 8M–Q), Siberian tiger (Figures 9A–C), clouded leopard (Figures 9S–V), humpback whale (Figures 10F–H), giraffe (Figures 10R–T), human (Figures 11D–F), and chimpanzee (Figures 11P–R). Morphologically, stellate neurons exhibited twisting dendrites that frequently approached the pial surface. Some appeared bipolar (Figure 10S) whereas others had multiple dendrites radiating in all directions (Figures 8A, 11D,P). Stellate neurons had 4.1 primary branches per neuron (ranging from 2.8 in the tiger to 5.6 in the elephant) with a dendritic plexus that generally appeared more complex in the human and elephant than in other species. Quantitatively, dendritic measures varied considerably, although they tended to be greater for the elephant, particularly for dendritic Vol and TDL (Figures 12A–D). Variation in each dendritic measure for stellate neurons across species was as follows: Vol = 14.63-fold, TDL = 4.02-fold, MSL = 2.14-fold, DSC = 3.56-fold, and Tor = 1.07-fold.

*Basket neurons* (Figure 3) were located in the lower third of the molecular layer (*Mean*_soma depth_ = 350 ± 134 μm). Their typically ovoid somata were larger than observed in stellate neurons, with a 3.86-fold difference in size across species (human = 113 μm^2^ < chimpanzee < leopard < tiger < whale < giraffe < manatee < elephant = 444 μm^2^). Sample tracings of basket neurons are provided for each species: African elephant (Figures 8D,E), Florida manatee (Figures 8R–V), Siberian tiger (Figures 9I–K), clouded leopard (Figures 9W–Y), humpback whale (Figures 10A–E), giraffe (Figures 10U–X), human (Figures 11A–C), and chimpanzee (Figures 11S–V). Morphologically, basket neurons were usually, but not always (Figures 8D, 11C), characterized by dendritic branches that extended laterally from the soma, travelling horizontally a short distance before curving toward the pial surface in a typical sea-fan shape (Figures 8E, 9X, 10D, 11S). Axons were visible in some neurons, allowing them to be traced over distances of several 100 μm (Figures 8R, 11C,U). These axons travelled transversely above the Purkinje cell layer and were sometimes observed to terminate in multiple pericellular nests (Figure 3F) with paintbrush tips (Figure 3E; Ramón y Cajal, 1909, 1911) around the somata of Purkinje cells. Basket neurons had an average of 4.1 primary dendrites (ranging from 3.0 in the whale to 6.1 in the manatee). As with stellate neurons, they appeared more dendritically complex in the human and elephant relative to the other species. Quantitatively, there was considerable variation among species, with the elephant generally exhibiting the largest dendritic values (Figures 12E–H). Ranges of variation in each dendritic measure for basket neurons across species were as follows: Vol = 13.51-fold, TDL = 4.83-fold, MSL = 1.72-fold, DSC = 3.10-fold, and Tor = 1.07-fold.

### Purkinje cell layer

*Purkinje neurons* were not quantified in the present sample because their dendritic complexity precluded (accurate) tracings; in fact, the distal dendritic plexuses in many of these neurons were completely black under the 60x objective. Nevertheless, sample photomicrographs (Figure 4) illustrate large, piriform somata from which the complex, prototypical two-dimensional dendritic plexus ascended throughout the molecular layer. These appeared morphologically similar across all species except the humpback whale (Figure 4C), where tertiary dendritic branches tended to ascend to the pial surface in straight, unbending manner. As such, the main dendritic branches of the humpback Purkinje neuron are much less convoluted than observed in the other species. This morphological difference is particularly clear when comparing the skeletal tracings of the humpback whale Purkinje neurons (Figures 4F,G) to those of the giraffe (Figures 4H,I), the other cetartiodactyl in the current study.

### Granule cell layer

*Lugaro neurons* (Figure 5) were usually located superficially in the granule cell layer immediately below the Purkinje cell bodies (*Mean*_soma depth_ = 543 ± 179 μm). Those Lugaro neurons located between Purkinje cell and granule cell layers usually possessed fusiform somata (e.g., Figures 8K, 10K, 11Z,AA); those deeper in the granule cell layer were more likely to possess triangular somata. In terms of soma size, these were the largest observed in the present sample, with a 5.01-fold difference in size across species (manatee = 270 μm^2^ < chimpanzee and giraffe < human < leopard < tiger < whale < elephant = 1354 μm^2^). There was an average of 3.9 primary dendrites per neuron (ranging from 3.2 in the tiger to 5.3 in the leopard). Those neurons with fusiform somata tended to be horizontally oriented in the parasagittal plane with dendrites that branched little and extended over several 100 μm (Figures 8K, 9L, 10J,K, 11G). Those with more triangular shaped somata were oriented in various directions, including perpendicular to the Purkinje cell layer, and were particularly common in the elephant (Figures 8J,K, 9N, 11Y). In contrast to other cerebellar neurons, which tended to be relatively uniform in appearance, the Lugaro neurons were more morphologically diverse, as indicated in the tracings for each species: African elephant (Figures 8I–L), Florida manatee (Figure 8W), Siberian tiger (Figures 9 L–N), clouded leopard (Figures 9Z–AA), humpback whale (Figures 10I–L), giraffe (Figures 10Y–AA), human (Figures 11G–I), and chimpanzee (Figure 11W–AA). Quantitatively, Lugaro neurons were disproportionately larger in the elephant than in any of the other species, particularly in terms of dendritic Vol (Figures 12I–L). Variation in each dendritic measure for Lugaro neurons across species was as follows: Vol = 25.72-fold, TDL = 3.36-fold, MSL = 2.66-fold, DSC = 3.44-fold, and Tor = 1.08-fold.

*Golgi neurons* (Figure 6) were also usually located superficially in the granule cell layer, although some were much deeper (*Mean*_soma depth_ = 512 ± 175 μm). They had irregular stellate, triangular, or polygonal somata, with a 3.05-fold difference in size across species (leopard = 242 μm^2^ < chimpanzee < human < tiger < whale < giraffe < elephant = 737 μm^2^). With an average of 6.5 primary dendrites per neuron (ranging from 5.3 in the whale to 8.2 in the elephant), they exhibited the highest number of primary dendrites in the present sample. These dendrites radiated relatively thick branches in all directions, forming a characteristic three-dimensional spherical field, as illustrated in Neurolucida tracings: African elephant (Figures 8F–H), Siberian tiger (Figures 9D–H), clouded leopard (Figures 9BB,CC), humpback whale (Figures 10P,Q), giraffe (Figures 10BB–EE), human (Figures 11J–L), chimpanzee (Figures 11GG–JJ). Quantitatively, as with most other neurons in the current sample, these achieved their greatest extent in the elephant (Figures 12M–P). Variation in each dendritic measure for Golgi neurons across species (except the manatee) was as follows: Vol = 12.94-fold, TDL = 3.89-fold, MSL = 1.76-fold, DSC = 4.77-fold, and Tor = 1.07-fold.

*Granule neurons* (Figure 7) were, on average, the most deeply located of all traced neurons (*Mean*_soma depth_ = 615 ± 181 μm), and the smallest, with a 1.36-fold difference in size across species (human = 50 μm^2^ < leopard < chimpanzee < whale and tiger < giraffe = 68 μm^2^). These were characterized by small, round cell bodies from which an average of 3.5 short dendrites emerged (ranging from 3.0 in the human to 3.8 in the giraffe, whale, and tiger), each terminating in gnarled, claw-like inflorescences. Axons, when visible, tended to ascend immediately toward the molecular layer (Figures 9Q, 10FF, 11M,BB,CC). Across species, there was little qualitative variation in granule neuron morphology: Siberian tiger (Figures 9O–R), humpback whale (Figures 10M–O), giraffe (Figures 10FF–HH), human (Figures 11M–O), and chimpanzee (Figures 11BB–FF). Quantitatively, granule neurons had the lowest median values of all dendritic measures except Tor, for which they exhibited the highest values (Figures 12Q–T). Variations in each dendritic measure for granule neurons across species (except the elephant and manatee) were as follows: Vol = 5.34-fold, TDL = 2.41-fold, MSL = 6.40-fold, DSC = 2.50-fold, and Tor = 1.29-fold.

### Sholl analyses

**Figure 13:**
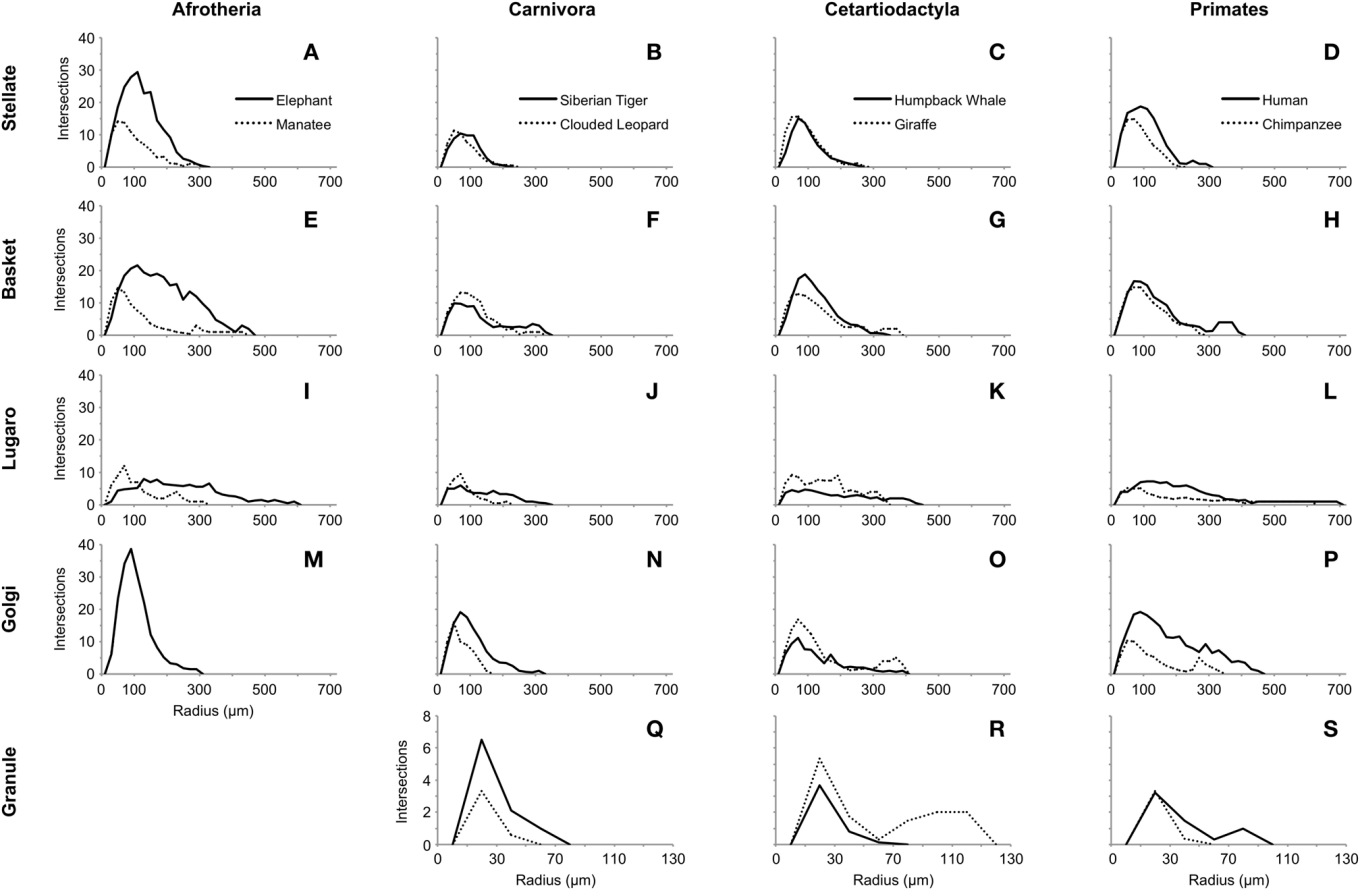
**Sholl analyses of five neuron types (stellate: A–D; basket: E–H; Lugaro: I–L; Golgi: M–P; granule: Q–S), arranged by taxonomic groupings (afrotherians: elephant and manatee; carnivores: tiger and clouded leopard; cetartiodactyls: humpback whale and giraffe; and primates: human and chimpanzee), indicating relative dendritic complexity of branching patterns**. Dendritic intersections were quantified at 20-μm intervals using concentric rings. Note that granule neurons in afrotherians, and Golgi neurons in the manatee are not illustrated here because they did not stain, and that the ordinate scale for granule neurons is much smaller than the scale for other neuron types.

Several limited observations can be made on the basis of the Sholl analyses (Figure 13). First, the peak in the number of intersections appeared to be around 100 μm from the soma for most neuron types for all species. Second, Lugaro neurons (Figures 13I–L) did not exhibit the same sharp peak in intersections as did other neurons; rather, they were relatively flat in their dendritic envelope, with dendrites that extended great distances from the soma, particularly in humans and elephants. Third, the elephant profile (Figures 13A,E,M) appeared markedly different (i.e., much higher peaks) from other species' profiles. Forth, granule neurons (Figures 13Q–S) had a much lower number of intersections than did other neurons, and exhibited a peak around 25 μm from the soma.

### Inferential statistical analyses across species

To examine species differences in dendritic measures, analyses proceeded with a third order MARSplines differentiation of species using TDL, MSL, DSC, Vol, Tor, and soma size. In brief, the procedure tested the dendritic measures and soma size of each neuron to assess if it could be identified as belonging to a particular species. Eight binary variables were created, one for each species. The analysis proceeded by utilizing a MARSplines model to test the hypothesis that species could be differentiated from each other based on just dendritic measures and soma size. As an example, to test the hypothesis with giraffes, a new attribute called giraffe was created, and it took a value 1 when the dendritic measures came from a giraffe neuron and a 0 when they did not. Eight of these binary (1/0) attributes were created, one for each species. Each of the 317 rows of neuronal data was coded with eight 1/0 attributes. For example, there was a row for a giraffe neuron with the eight new species attributes arranged in alphabetical order (chimpanzee, clouded leopard, elephant, giraffe, human, humpback whale, manatee, tiger); then, these attributes were coded 0, 0, 1, 0, 0, 0, 0, 0, respectively. Next, the MARSplines analysis was used with the giraffe attribute as the dependent measure and the dendritic measurements as the independent measures to test the null hypothesis by comparing neuronal measures contributed by the giraffe to those contributed by the other seven species. In the present study, the null hypothesis was that there was no relationship between the dendritic measures and species, or, in other words, that the dendritic measures for a given neuron could not be assigned reliably to a given species. The null hypothesis was rejected at either *p* < 0.10 on the *F*-test or a correct prediction (≥0.90) about whether a neuron did or did not belong to a particular species. Results were represented as counts (i.e., number of neurons that successfully differentiated a given species from all the other species) and percentages of correct and incorrect species assignments, an *r*^2^ statistic, and an *F* statistic. *F* statistics were produced using each binary species attribute as the categorical or class measure and the “is or isn't” as the target species score estimated from the MARSplines. The estimated scores were continuous, and the cut point for whether or not the estimated value was the species under analysis or some other species was 0.5. This cut point was used in calculating the percentage of correct predictions. That is, if giraffes were being evaluated, the observed value of the giraffe 1/0 attribute would have been 1 and if the estimated value of giraffe was ≥0.5, we would conclude that the MARSpline equation correctly differentiated giraffes from all other species in that row of data.

The null hypothesis of no relationship between dendritic measures and species was rejected for all species because all *F* statistics were significant at *p* ≤ 0.01: elephant [*F*_(1, 253)_ = 8097.30, *r*^2^ = 0.967], manatee [*F*_(1, 267)_ = 334.33, *r*^2^ = 0.526], tiger [*F*_(1, 315)_ = 137.93, *r*^2^ = 0.265], leopard [*F*_(1, 315)_ = 110.63, *r*^2^ = 0.260], whale [*F*_(1, 315)_ = 373.76, *r*^2^ = 0.208], giraffe [*F*_(1, 315)_ = 290.12, *r*^2^ = 0.448], human [*F*_(1, 315)_ = 196.05, *r*^2^ = 0.349], and chimpanzee [*F*_(1, 315)_ = 720.62, *r*^2^ = 0.443]. Further, as noted in the correct-incorrect confusion matrices (Table 4), the percentage of correct predictions for neuronal fit to a particular species ranged from 85.5% in the chimpanzee to 99.6% in the elephant. To elaborate, in the elephant, 19 of 20 neurons were correctly identified as belonging to the elephant, and 235 of 235 were correctly identified as not belonging to the elephant (thus, 99.6%). In the chimpanzee, 63 of 86 neurons were correctly identified as belonging to the chimpanzee, and 208 of 231 were correctly identified as not belonging to the chimpanzee (thus, 85.5%). What these results indicate is that dendritic measures and soma size were accurate predictors of each species in the current sample because these measures, taken together, allowed neurons to be correctly identified as belonging to a particular species.

**Table 4:**
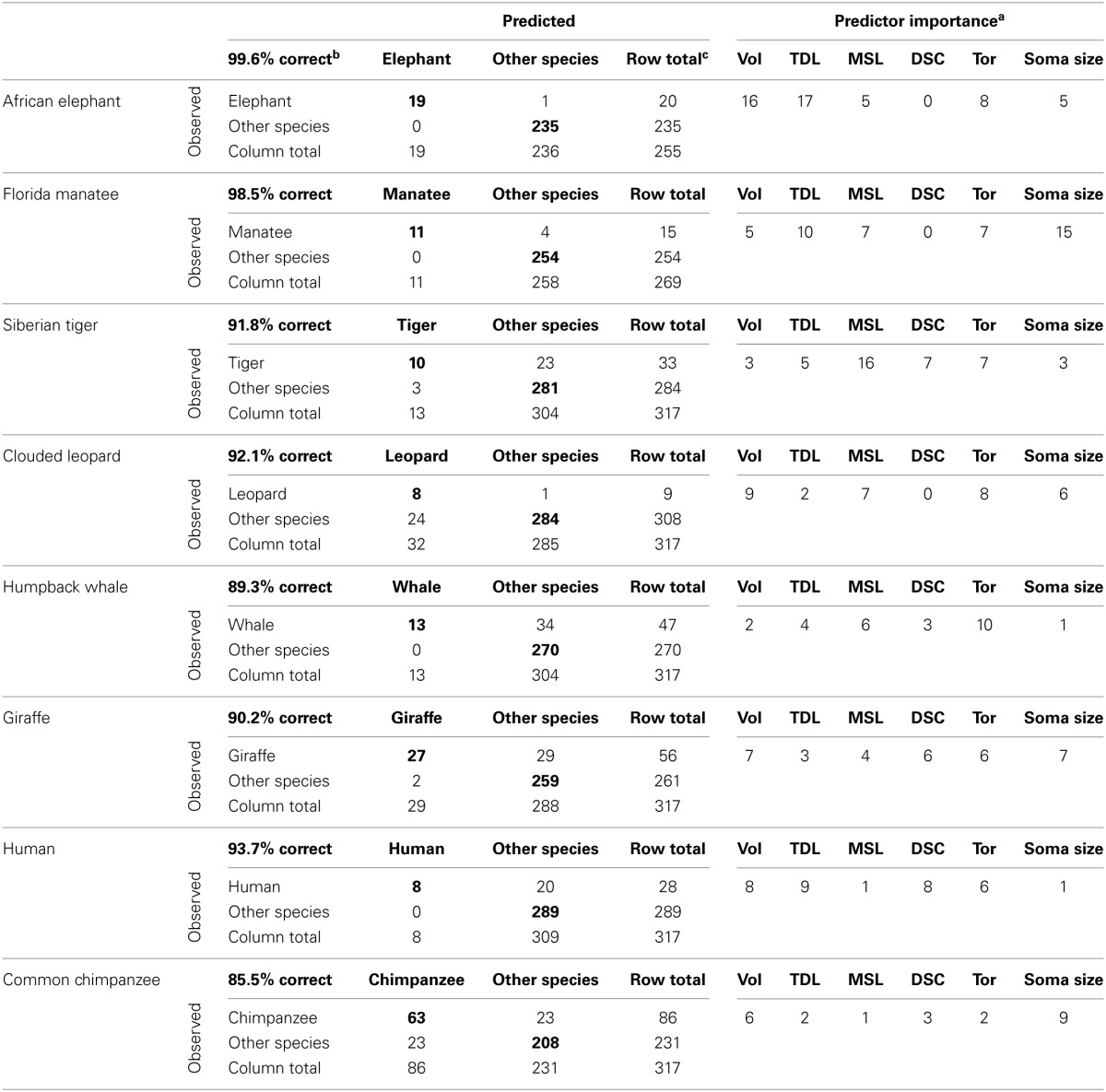
**Correct-incorrect confusion matrices for differentiation of species**.

*^a^Predictor importance indicates how many times each measure appears in the regression analysis for that particular species. See text for more details*.

*^b^Bold numbers represent the correct predictions. The percentage correct is calculated by dividing the sum of the two bold numbers by the total number of neurons examined. So, for the elephant: (19 + 235)/255 = 0.996*.

*^c^Note that the total number of neurons for elephants and manatees is not 317, as for all other species, because some neuron types did not stain in the two afrotherians*.

Further, the procedure provided the relative importance of each attribute (i.e., dendritic Vol, TDL, soma size, etc.) in determining whether a neuron belonged or did not belong to a particular species (Table 4). These measures indicated the number of times each attribute or predictor was used in the equations testing the null hypothesis for each species. In this framework, for example, an attribute with three appearances in the analysis for a given species would be three times more important to the prediction than an attribute with only one appearance. As noted in Table 4, across all species, dendritic Vol, Tor, and TDL appeared to be the overall most important (i.e., most utilized by the analysis) measures for differentiating each species from the others, whereas DSC was the least important. However, the combination of these variables was unique for each species. For example, in the elephant, TDL (17 appearances) and dendritic Vol (16 appearances) were the most influential measures in species identification; in the chimpanzee, however, soma size (9 appearances) and dendritic Vol (6 appearances) were the most important predictors.

## Discussion

The present study contributes to a limited database of comparative neuroanatomy (Manger et al., 2008) by examining cerebellar neuronal morphology across a wide variety of large brained mammals both qualitatively and quantitatively. Although the current sample exhibited a large range in cerebellar volume, the overall volume fraction of the cerebellum (13.6 ± 3.1%) is consistent with that reported by Clark et al. (2001) across 9 mammalian taxa, namely 13.5 ± 2.4%. There was considerable uniformity across species in terms of histology insofar as the cerebellar cortex followed the trilaminate pattern typical of birds and mammals (Ramón y Cajal, 1909, 1911; Sultan and Glickstein, 2007). In terms of morphology, each neuronal type within the cerebellar cortex was generally consistent across the eight species. Quantitatively, however, there was substantial species variation in dependent dendritic measures for each neuronal type, with neurons in the elephant tending to be larger than those in other species for most measures. Finally, inferential analyses detected significant species differences in dendritic measures and soma size.

### Methodological considerations

General constraints pertaining to Golgi-stained materials have been extensively outlined elsewhere (Jacobs and Scheibel, 2002; Jacobs et al., 2011). These include (1) characteristics of incomplete impregnations (Williams et al., 1978; Braak and Braak, 1985), (2) the effects of post-mortem delay and suboptimal fixation (de Ruiter, 1983; Jacobs and Scheibel, 1993; Jacobs et al., 1993, 2001; Friedland et al., 2006), and (3) the relative merits of the Golgi stain compared to other histological techniques (Scheibel and Scheibel, 1978; Buell, 1982; Ohm and Diekmann, 1994; Jacobs et al., 1997). Another inherent limitation in Golgi studies is the effect of section thickness on estimations of dendritic extent (Jacobs et al., 1997). Larger neurons (such as those in the elephant) are more affected by sectioning, resulting in an attenuation of dendritic measures. In the present study, this means that actual differences among species are probably larger than the data suggest. Thus, the present dendritic measurements should be seen as representing *relative* rather than absolute values. To completely eliminate cut dendrites would require (1) tissue sections ~1000 μm thick, which would make them completely opaque, or (2) tracing cut dendritic segments across serial sections, a technique that is not accurate or feasible in a Golgi study this extensive, where multiple, overlapping neural processes appear in any given section of tissue. Finally, neurons in the present study were classified based solely on somatodendritic architecture and their relative location within the cerebellar cortex, which is typical for Golgi impregnations. It was not possible to further subcategorize neuron types based on axonal plexi distributions (Bishop, 1993; Lainé and Axelrad, 1996), lipofuscin pigmentation (Braak and Braak, 1983), or immunohistochemistry and neurochemical phenotypes (Lainé and Axelrad, 2002; Simat et al., 2007).

### Neuronal morphology: qualitative observations

In general, neurons in the present sample were similar in morphology to those described in primates (Fox et al., 1967; Rakic, 1972; Braak and Braak, 1983; Mavroudis et al., 2013) and rodents (O'Leary et al., 1968; Chan-Palay and Palay, 1972; Palay and Chan-Palay, 1974). For example, molecular layer interneurons in the present sample had hemielipsoid dendritic systems that greatly resembled those described by Rakic (1972), with deeper neurons having an ascending dendritic plexus, intermediate neurons having a dendritic plexus extending in all directions, and superficial neurons having mostly descending dendritic branches. The axonal ramifications observed in basket neurons were also similar to those described in monkeys (Fox et al., 1967), cats (Bishop, 1993), and rats (Palay and Chan-Palay, 1974). There were, however, a few morphological observations of particular interest. First, the Lugaro neurons in most of the current species resembled those described in the literature (Christ, 1985; Melik-Musyan and Fanardzhyan, 1998, 2004). They also resembled those identified by Braak and Braak (1983) in humans as Type II neurons despite Braak and Braak stating that these types of cells may be displaced basket neurons, a finding disputed by others (Lainé and Axelrad, 1996). In contrast, many Lugaro neurons in the elephant appeared distinctive because of their vertical orientation and idiosyncratic dendritic arrangements. These were unusual in the present sample, although they have been briefly described in the cat (Sahin and Hockfield, 1990; Melik-Musyan and Fanardzhyan, 1998) and the duck (O'Leary et al., 1968). Second, most of the traced Golgi neurons resembled those observed in the literature, and are consistent with Braak and Braak's (1983) Type I neuron designation. However, there are some (e.g., Figures 8G, 11K) that resembled Braak and Braak's Type III description (see their Figure 7) insofar as they exhibited, among other characteristics, a very dense dendritic arborization that extended into the molecular layer.

Purkinje neurons were remarkably similar in their basic morphology across most species examined, and were consistent with those described in other species (rat: Roth and Häuser, 2001; Sawada et al., 2010; guinea-pig: Rapp et al., 1994). The notable exception was the Purkinje neuron in the humpback whale, which exhibited straighter, more vertically oriented tertiary dendritic arbors than any of the other species examined. This dendritic pattern differed from the other aquatic mammal in the current study (e.g., the manatee) and from the other cetartiodactyl (e.g., the giraffe). Humpback whale Purkinje neurons actually resembled those observed in mormyrid electric fish (Meek and Nieuwenhuys, 1991; Meek, 1992; Han et al., 2006), with a palisade pattern of relatively unbranched, molecular layer dendritic arbors that extend in parallel to the pial surface (Nieuwenhuys and Nicholson, 1967; Meek and Nieuwenhuys, 1991). Visual observation of our Golgi stains suggests, however, that the humpback whale may have substantially fewer palisade dendrites than the ~50 noted in mormyrid Purkinje neurons (Meek and Nieuwenhuys, 1991). Direct comparison within cetaceans is problematic because only Adanina (1965) provides any images of Golgi impregnated cerebellar cortical neurons in cetaceans (specifically, *Tursiops truncatus* and *Delphinus delphis*). Adanina suggests that there is heterogeneity in the somata of Purkinje neurons, with some being pear-shaped and others being fusiform. Unfortunately, although Purkinje neuron dendritic arbors in the dolphin may be consistent with our observations in the humpback whale, Adanina's impregnation is insufficient for a definitive conclusion. Further research is necessary to confirm whether Purkinje neurons in mysticetes are morphologically different from those in other cetaceans, or from mammals in general.

### Neuronal morphology: quantitative observations

With the exception of Purkinje neuron reconstructions and a few measurements of individual dendritic segments, there appear to be very limited quantitative dendritic data on cerebellar cortical neurons (Palay and Chan-Palay, 1974; Braak and Braak, 1983). There is, however, a small number of digital reconstructions of molecular layer interneurons (classical stellate and basket neurons) in the rat based on rapid Golgi impregnations (Sultan and Bower, 1998). Not surprisingly, soma size (69 μm^2^) in these rat neurons was smaller than in stellate neurons from all species in the present sample. The number of primary branches (3.1) in the rat was also near the minimum of the current sample (tiger: 2.8 primary branches in stellate neurons; whale: 3.0 primary branches in basket neurons). Similarly, the dendritic length for rat molecular interneurons (1189 μm) was shorter than the lowest values for stellate (leopard TDL = 1367 μm) and basket neurons (tiger TDL = 1285 μm) in the current sample (see Figure 12), although it should be noted that Sultan and Bower (1998) sectioned their tissue at 100 μm rather than 120 μm, which could result in more attenuated dendritic length values. To the extent that we can generalize from the present sample, it appears that, among molecular layer interneurons, the more superficial stellate neurons tended to be smaller than the deeper basket neurons for most dendritic measures, a finding consistent with observations in the cerebral neocortex, where deeper neurons tend to be larger than more superficial neurons (Jacobs et al., 1997, 2001). In the granule cell layer, Lugaro and Golgi neurons were typically similar in overall size (e.g., dendritic Vol and TDL); however, they exhibited vastly different morphologies, as reflected in typically greater MSL values for Lugaro neurons (indicating long, unbranched dendrites) and higher DSC values for Golgi neurons (suggesting a more complex dendritic branching pattern).

Variability in neuronal measures across species was much smaller than that observed for brain mass (64-fold) and cerebellar volume (103-fold). In general, dendritic measurements and soma size tended to be positively correlated with cerebellar volume for most neuron types, although this tendency was skewed by the large size of elephant neurons. There was a 3.08-fold difference in soma size among the species in the current sample, with the elephant having the largest values across all neuron types. Moreover, most neuron types were characterized by a 3.5- to 5-fold range of variation in dendritic measures across species. The Lugaro neuron, however, averaged a 7.25-fold variation across species, mainly because of the extraordinary size of Lugaro neurons in the elephant (Maseko et al., 2012a)—note that excluding the elephant data resulted in a 3.19-fold variation in Lugaro neurons. Although the length of Lugaro dendrites in the present sample appears to be within the range of what has been reported in rats (i.e., from 100 to 700 μm from the soma; Lainé and Axelrad, 1996), it is the measure of Lugaro dendritic Vol. that especially differentiates the elephant from other species. For dendritic Vol, there was an average 14.43-fold increase across species in the current sample, as opposed to an average 2.81-fold increase for all other dendritic measures. This suggests that dendritic Vol might scale more steeply than other dendritic measures for cross-species comparisons in the cerebellum, an observation that seems to have been confirmed in the current MARSplines analysis, which indicated that dendritic Vol was the most consistently used variable for interspecies differentiation. These findings also appear consistent with the suggestion that there is a positive relationship between brain mass and dendritic extent in the neocortex (Elston et al., 2006; Herculano-Houzel et al., 2006; Sarko et al., 2009; Jacobs et al., 2011; Manger et al., 2013), a corollary being that, similar to the cerebral cortex (Haug, 1967, 1987), neuronal density in the cerebellum appears to be inversely related to brain mass (Lange, 1975; Maseko et al., 2012a).

### Inferential statistical comparisons across species

One goal of the present investigation was to compare neuronal morphology in the cerebellar cortex across several large brained mammals not previously examined. We tested for species differences using MARSplines analyses, which indicated that there were significant differences in dendritic measures (and soma size) among all species. Moreover, this analysis revealed not only which measures were most important for differentiating individual species, but also the unique combinations and weightings of these measures (Table 4). In future studies, a data set with a much larger number of neurons, and with all neuron types represented for every species, would enable a more detailed evaluation of the relative importance of neuron types (e.g., Lugaro vs. Golgi) to species differentiation. At this point, an interesting question is whether the evolutionary, ecological, and behavioral adaptations that influence brain mass and cerebellar volume, might also shape aspects of the somatodendritic morphology in neurons themselves.

### Functional speculations

The large-brained mammals in the current sample represent a diverse range of ecological, somatic, and behavioral adaptations. Here, we can only speculate very generally how these adaptations may relate to factors such as cerebellar volume and neuronal morphology in these species. Insofar as the cerebellum has traditionally been implicated in motor control (Fulton and Dow, 1937; Marr, 1969; Glickstein and Yeo, 1990), the motor system of a particular species is often the initial focal point (Onodera and Hicks, 1999). For example, the elephant possesses the largest absolute and relative cerebellar volume of any mammal investigated to date (Shoshani et al., 2006; Maseko et al., 2012b), a finding typically explained with reference to the fine motor control demands of its trunk (Endo et al., 2001; Maseko et al., 2012b). A more integrative, and perhaps parsimonious, perspective suggests that the cerebellum is not involved exclusively with motor control, but rather that it is engaged in monitoring and adjusting the acquisition of sensory information for the rest of the nervous system (Bower, 1992, 1997; Gao et al., 1996). As such, one factor contributing to the large elephant cerebellum may be the documented importance of the trunk in multi-sensory exploration of the environment (Rasmussen and Munger, 1996; Bagley et al., 2006; Foerder et al., 2011). If this theory is extended to the domain of communication, the auditory-tactile infrasound information perceived through the elephant's feet may also contribute to its enlarged cerebellum (Garstang, 2004; Bouley et al., 2007; Soltis, 2009).

Additional investigations into the sensory role of cerebellum may provide insight into the other species examined in the current study. For example, imaging research has revealed that the lateral cerebellar hemispheres are involved in sensory acquisition and discrimination in humans (Parsons et al., 1997), a finding that may also apply to chimpanzees. Certainly, this is consistent with the expansion of the lateral cerebellum in hominoids relative to other primates (Rilling and Insel, 1998; MacLeod et al., 2003; Rilling, 2006). Electrophysiological research has shown that cats have larger tactile representations of the forelimbs in the lateral cerebellar hemispheres than do rodents (e.g., mice, rats, and guinea pigs) because cats use their forelimbs more for sensory exploration of their environment than do rodents, which depend more on tactile information from the face region (Welker, 1964; Bower, 1997, 2011). Speculatively, cerebellar sensory representations for the manatee may resemble those of rodents insofar as the manatee has a sensitive, perioral tactile system for exploring its aquatic environment (Marshall et al., 1998, 2003; Reep et al., 2001). In contrast, the felines of the current study may be characterized by strong forelimb representation in the lateral cerebellum. Finally, such a sensory focus on cerebellar processing may help clarify why there is a relative increase in cerebellar volume in microchiropterans and odontocete cetaceans (Baron et al., 1996; Marino et al., 2000) vis-à-vis primates (Maseko et al., 2012b). From the perspective of motor control, this observation is difficult to explain insofar as primates arguably have greater fine motor dexterity (Darian-Smith et al., 2007; Kaas, 2008). However, both microchiropterans and odontocetes rely extensively on the coordinated use of sensory surfaces when exploring their environment with echolocation (Norris et al., 1961; Ghose et al., 2006; Surlyke et al., 2009; Akamatsu et al., 2010), and this may contribute to an expansion in cerebellar tissue. By extension, the humpback whale cerebellum may also be affected by the whale's extensive vocal repertoire (Mercado et al., 2010; Garland et al., 2011) which, similar to echolocation, may serve as a type of sonar that provides sensory information about its aquatic world (Frazer and Mercado, 2000).

Finally, although the basic circuitry of the cerebellar cortex is fairly well documented in a limited number of species, discerning structure-function relationships can be challenging (Sultan and Glickstein, 2007; Schilling et al., 2009). This is especially true when direct electrophysiological experimentation on a species is not possible. There are, however, two morphological findings of particular functional interest in the present study: (1) the distinctive morphology and large size of Lugaro neurons in the elephant cerebellum, and (2) the presence of a palisade dendritic pattern for Purkinje neurons in the humpback whale. With regards to first observation, what remains unclear is whether the elephant Lugaro neurons are functionally connected in the same manner as demonstrated in other species, that is, whether they receive serotonergic input (Dieudonné and Dumoulin, 2000; Geurts et al., 2003), input from Purkinje neuron collaterals (Lainé and Axelrad, 1996; Geurts et al., 2003), and/or whether they project to molecular layer interneurons (Flace et al., 2004; Ambrosi et al., 2007) and to Golgi neurons (Melik-Musyan and Fanardzhyan, 1998; Dumoulin et al., 2001; Crook et al., 2006). The expansive dendritic arbors of elephant Lugaro neuorons would suggest a broad sampling of local input but, because we have no information on their axonal projections, it is unclear to what extent they exert inhibitory feedback on Purkinje neurons, modulate mossy fiber input, and/or contribute to long-term depression (Geurts et al., 2003; Melik-Musyan and Fanardzhyan, 2004). With regards to the humpback whale Purkinje neuron dendrites, any functional speculation would be premature until future research confirms the current, tentative findings. If such Purkinje cell dendritic morphology actually obtains in mysticetes, or in cetaceans in general, then the next question would be whether the acoustic world of cetaceans and the electrosensory system in mormyrids have any neurofunctional commonalities. Only more detailed comparative research will address such issues.

### Conflict of interest statement

The authors declare that the research was conducted in the absence of any commercial or financial relationships that could be construed as a potential conflict of interest.

## References

[B1] Adanina V. (1965). Neurons and connections of the flocculus in dolphins. Arkh. Anat. 56, 29–35 5364902

[B2] Akamatsu T. Wang D. Wang K. Li S. Dong S. (2010). Scanning sonar of rolling porpoises during prey capture dives. J. Exp. Biol. 213, 146–152 10.1242/jeb.037655 20008371

[B3] Altman J. Bayer S. A. (1977). Time of origin and distribution of a new cell type in the rat cerebellar cortex. Exp. Brain Res. 29, 267–274 10.1007/BF00237046 913518

[B4] Ambrosi G. Flace P. Lorusso L. Girolamo F. Rizzi A. Bosco L. (2007). Non-traditional large neurons in the granular layer of the cerebellar cortex. Eur. J. Histochem. 51, 59–64 17703595

[B5] Anderson K. Bones B. Robinson B. Hass C. Lee H. Ford K. (2009). The morphology of supragranular pyramidal neurons in the human insular cortex: a quantitative Golgi study. Cereb. Cortex 19, 2131–2144 10.1093/cercor/bhn234 19126800

[B6] Anderson K. Yamamoto E. Kaplan J. Hannan M. Jacobs B. (2010). Neurolucida Lucivid vs. Neurolucida camera: a quantitative and qualitative comparison of three-dimensional neuronal reconstructions. J. Neurosci. Methods 186, 209–214 10.1016/j.jneumeth.2009.11.024 19963008

[B7] Bagley K. R. Goodwin T. E. Rasmusen L. E. L. Schulte B. A. (2006). Male African elephants, *Loxodonta africana*, can distinguish oestous status via urinary signals. Animal Behav. 71, 1439–1445 10.1016/j.anbehav.2006.01.003

[B8] Baron G. Stephan H. Frahm H. D. (1996). Comparative Neurobiology in Chiroptera. Basel: Birkhauser Verlag

[B9] Bell B. A. Ferron J. M. Kromrey J. D. (2008). Cluster size in multilevel models: the impact of sparse data structures on point and interval estimates in two-level models, in JSM Proceedings, Section on Survey Research Methods, 1122–1129 Available Online at: http://www.amstat.org/Sections/Srms/Proceedings/y2008/Files/300933.pdf

[B10] Berk R. A. Freedman D. A. (2003). Statistical assumptions as empirical commitments, in Punishment and Social Control: Essays in Honor of Sheldon L. Messinger, eds Cohen S. Blomberg T. G. (New Brunswick, NJ: Aldine de Gruyter), 235–254

[B11] Bishop G. A. (1993). An analysis of HRP-filled basket cell axons in the cat's cerebellum. I. Morphometry and configuration. Anat. Embrol. 188, 287–297 8250283 10.1007/BF00188219

[B12] Bok S. T. (1959). Histonomy of the Cerebral Cortex. Amsterdam: Elsevier

[B13] Bolk L. (1906). Das Cerebellum der Säugetiere: eine Vergleichende Anatomische Untersuchung. Haarlem: Fischer

[B14] Bouley D. M. Alarcon C. N. Hildebrandt T. O'Connell-Rodwell C. E. (2007). The distribution, density and three-dimensional histomorphology of Pacinian corpuscles in the foot of the Asian elephant (*Elephas maximus*) and their potential role in seismic communication. J. Anat. 211, 428–435 10.1111/j.1469-7580.2007.00792.x 17711421 PMC2375831

[B15] Bower J. M. (1992). Is the cerebellum a motor control device? Behav. Brain Sci. 15, 714–715

[B16] Bower J. M. (1997). Is the cerebellum sensory for motor's sake, or motor for sensory's sake: the view from the whiskers of a rat? Prog. Brain Res. 114, 483–516 10.1016/S0079-6123(08)63381-6 9193161

[B17] Bower J. M. (2011). Functional implications of tactile projection patterns to the lateral hemispheres of the cerebellum of the albino rat: the legacy of Wally Welker. Ann. N.Y. Acad. Sci. 1225, 130–141 10.1111/j.1749-6632.2011.06020.x 21535000

[B18] Braak E. Braak H. (1983). On three types of large nerve cells in the granular layer of the human cerebellar cortex. Anat. Embryol. 166, 67–86 10.1007/BF00317945 6837933

[B19] Braak H. Braak E. (1985). Golgi preparations as a tool in neuropathology with particular reference to investigations of the human telencephalic cortex. Prog. Neurobiol. 25, 93–139 10.1016/0301-0082(85)90001-2 2418465

[B20] Breathnach A. S. (1955). The surface features of the brain of the humpback whale (*Megaptera novaeangliae*). J. Anat. 89, 343–352 13251964 PMC1244762

[B21] Buell S. J. (1982). Golgi-Cox and rapid Golgi methods as applied to autopsied human brain tissue: widely disparate results. J. Neuropathol. Exp. Neurol. 41, 500–507 10.1097/00005072-198209000-00003 6180137

[B22] Calvet M.-C. Calvet J. (1984). Computer assisted analysis of HRP labeled and Golgi stained Purkinje neurons. Prog. Neurobiol. 23, 251–272 10.1016/0301-0082(84)90006-6 6398453

[B23] Chan-Palay V. Palay S. L. (1970). Interrelations of basket cell axons and climbing fibers in the cerebellar cortex of the rat. Z. Anat. Entwickl-Gesch. 131, 191–227 10.1007/BF00523377 5490536

[B24] Chan-Palay V. Palay S. L. (1972). The stellate cells of the rat's cerebellar cortex. Z. Anat. Entwickl-Gesch. 136, 224–248 10.1007/BF00519180 5042759

[B25] Christ H. (1985). Fusiform nerve cells of the granular layer in the cerebellar cortex of the baboon. Neurosci. Lett. 56, 195–198 10.1016/0304-3940(85)90128-4 4011056

[B26] Clark D. A. Mitra P. P. Wang S. S.-H. (2001). Scalable architecture in mammalian brains. Nature 411, 189–193 10.1038/35075564 11346794

[B27] Crook J. Hendrickson A. Robinson F. R. (2006). Co-localization of glycine and GABA immuoreactivity in interneurons in macaca monkey cerebellar cortex. Neuroscience 141, 1951–1959 10.1016/j.neuroscience.2006.05.012 16784818

[B28] Darian-Smith I. Galea M. P. Darian-Smith C. (2007). Manual dexterity: how does the cerebral cortex contribute? Clin. Exp. Pharmacol. Physiol. 23, 948–956 10.1111/j.1440-1681.1996.tb01147.x 8911739

[B29] Dean P. Porrill J. Ekerot C.-F. Jörntell H. (2010). The cerebellar microcircuit as an adaptive filter: experimental and computational evidence. Nat. Rev. Neurosci. 11, 30–43 10.1038/nrn2756 19997115

[B30] Dell L.-H. Patzke N. Bhagwandin A. Bux F. Fuxe K. Barber G. (2012). Organization and number of orexinergic neurons in the hypothalamus of two species of Cetartiodactyla: a comparison of giraffe (*Giraffa camelopardalis*) and harbour porpoise (*Phocoena phocoena*). J. Chem. Neuroanat. 44, 98–109 10.1016/j.jchemneu.2012.06.001 22683547 PMC3551539

[B31] de Ruiter J. P. (1983). The influence of post-mortem fixation delay on the reliability of the Golgi silver impregnation. Brain Res. 266, 143–147 10.1016/0006-8993(83)91317-3 6189557

[B32] Dieudonné S. Dumoulin A. (2000). Serotonin-driven long-range inhibitory connections in the cerebellar cortex. J. Neurosci. 20, 1837–1848 10684885 10.1523/JNEUROSCI.20-05-01837.2000 PMC6772906

[B33] Dumoulin A. Triller A. Dieudonné S. (2001). IPSC kinetics at identified GABAergic and mixed GABAergic and glycinergic synapses onto cerebellar Golgi cells. J. Neurosci. 21, 6045–6047 11487628 10.1523/JNEUROSCI.21-16-06045.2001 PMC6763194

[B34] Elston G. N. Benavides-Piccione R. Elston A. Zietsch B. Defelipe J. Manger P. (2006). Specializations of the granular prefrontal cortex of primates: implications for cognitive processing. Anat. Rec. 288, 26–35 10.1002/ar.a.20278 16342214

[B35] Endo H. Hayashi Y. Komiya T. Narushima E. Sasako M. (2001). Muscle architecture of the elongated nose in the Asian elephant *(Elephas maximus)*. J. Vet. Med. Sci. 63, 533–537 10.1292/jvms.63.533 11411499

[B36] Flace P. Benagiano V. Lorusso L. Girolamo F. Rizzi A. Virgintino D. (2004). Glutamic acid decarboxylase immunoreative large neurons types in the granular layer of the human cerebellar cortex. Anat. Embrol. 208, 55–64 10.1007/s00429-003-0374-x 15014985

[B37] Foerder P. Galloway M. Barthel T. Moore D. E. III. Reiss D. (2011). Insightful problem solving in an Asian elephant. PLoS ONE 6:e23251 10.1371/journal.pone.0023251 21876741 PMC3158079

[B38] Foster R. E. Peterson B. E. (1986). The inferior olivary complex of guinea pig: cytoarchitecture and cellular morphology. Brain Res. Bull. 17, 785–800 10.1016/0361-9230(86)90090-0 3801935

[B39] Fox C. A. Hillman D. E. Siegesmund K. A. Dutta C. R. (1967). The primate cerebellar cortex: a Golgi and electron microscopic study. Prog. Brain Res. 25, 174–225 10.1016/S0079-6123(08)60965-6 4866553

[B40] Frazer L. N. Mercado E. 3rd. (2000). A sonar model for humpback whale song. IEEE J. Ocean. Eng. 25, 160–182 10.1109/48.820748

[B41] Freedman D. A. (2004). Sampling in the Encyclopedia of Social Science Research Methods, Vol. 3, eds Lewis-Beck M. Bryman A. Liao T. F. (Thousand Oaks, CA: Sage Publications), 986–990

[B42] Friedland D. R. Los J. G. Ryugo D. K. (2006). A modified Golgi staining protocol for use in the human brain stem and cerebellum. J. Neurosci. Methods 150, 90–95 10.1016/j.jneumeth.2005.06.004 16081162

[B43] Friedman J. H. (1991). Multivariate adaptive regression splines. Ann Stat 19, 1–67 10.1214/aos/1176347963 8548103

[B44] Fulton J. F. Dow R. S. (1937). The cerebellum: a summary of functional localization. Yale J. Biol. Med. 10, 89–119 21433753 PMC2601804

[B45] Gao J. H. Parsons L. M. Bower J. M. Xiong J. Li J. Fox P. T. (1996). Cerebellum implicated in sensory acquisition and discrimination rather than motor control. Science 272, 545–547 10.1126/science.272.5261.545 8614803

[B46] Garland E. C. Goldizen A. W. Rekdahl M. L. Constantine R. Garrigue C. Hauser N. D. (2011). Dynamic horizontal cultural transmission of humpback whale song at the ocean basin scale. Curr. Biol. 21, 687–691 10.1016/j.cub.2011.03.019 21497089

[B47] Garstang M. (2004). Long-distance, low-frequency elephant communication. J. Comp. Physiol. A. Neuroethol. Sens. Neural Behav. Physiol. 190, 791–805 10.1007/s00359-004-0553-0 15349746

[B48] Geurts F. J. Schutter E. D. Dieudonné S. (2003). Unravelling the cerebellar cortex: cytology and cellular physiology of large-sized interneurons in the granular layer. Cerebellum 2, 290–299 10.1080/14734220310011948 14964688

[B49] Ghose K. Horiuchi T. K. Krishnaprasad P. S. Moss C. F. (2006). Echolocating bats use a nearly time-optimal strategy to intercept prey. PLoS Biol. 4:e108 10.1371/journal.pbio.0040108 16605303 PMC1436025

[B50] Glickstein M. Yeo C. (1990). The cerebellum and motor learning. J. Cog. Neurosci. 2, 69–80 10.1162/jocn.1990.2.2.69 23972018

[B51] Golgi C. (1874). Sulla fina anatomia del cevelletto umano. Arch. Ital. per le Mal. Nerv. 2, 90–107

[B52] Halavi M. Hamilton K. A. Parekh R. Ascoli G. A. (2012). Digital reconstructions on neuronal morphology: three decades of research trends. Front. Neurosci. 6:49 10.3389/fnins.2012.00049 22536169 PMC3332236

[B53] Han V. Z. Meek J. Campbell H. R. Bell C. C. (2006). Cell morphology and circuitry in the central lobes of the mormyrid cerebellum. J. Comp. Neurol. 497, 309–325 10.1002/cne.20983 16736465

[B54] Hastie T. Tibshirani R. Friedman J. (2009). The Elements of Statistical Learning: Data Mining, Inference, and Prediction, 2nd Edn. New York, NY: Springer-Verlag 10.1007/978-0-387-84858-7

[B55] Haug H. (1967). Zytoarchitektonische Untersuchungen an der Hinrnrinde des Elefanten. Anat. Anz. 120, 331–337 6001125

[B56] Haug H. (1987). Brain sizes, surfaces, and neuronal sizes of the cortex cerebri: a stereological investigation of man and his variability and a comparison with some mammals (primates, whales, marsupials, insectivores, and one elephant). Am. J. Anat. 180, 126–142 10.1002/aja.1001800203 3673918

[B58] Herculano-Houzel S. Mota B. Lent R. (2006). Cellular scaling rules for rodent brains. Proc. Natl. Acad. Sci. U.S.A. 103, 12138–12143 10.1073/pnas.0604911103 16880386 PMC1567708

[B59] Iwaniuk A. N. Hurd P. L. Wylie D. R. W. (2006). The comparative morphology of the cerebellum in Caprimulgiform birds: evolutionary and functional implications. Brain Behav. Evol. 67, 53–68 10.1159/000089120 16244465

[B60] Jacobs B. Lubs J. Hannan M. Anderson K. Butti C. Sherwood C. C. (2011). Neuronal morphology in the African elephant (*Loxodonta africana*) neocortex. Brain Struct. Funct. 215, 273–298 10.1007/s00429-010-0288-3 21079992

[B61] Jacobs B. Schall M. Scheibel A. B. (1993). A quantitative dendritic analysis of Wernicke's area in humans. II. Gender, hemispheric, and environmental factors. J. Comp. Neurol. 327, 97–111 10.1002/cne.903270108 8432910

[B136] Jacobs B. Driscoll L. Schall M. (1997). Life-span dendritic and spine changes in areas 10 and 18 of human cortex: a quantitative Golgi study. J. Comp. Neurol. 386, 661–680 10.1002/(SICI)1096-9861(19971006)386:4<661::AID-CNE11>3.0.CO;2-N 9378859

[B137] Jacobs B. Schall M. Prather M. Kapler E. Driscoll L. Baca S. (2001). Regional dendritic and spine variation in human cerebral cortex: a quantitative Golgi study. Cereb. Cortex 11, 558–571 10.1093/cercor/11.6.558 11375917

[B62] Jacobs B. Scheibel A. B. (1993). A quantitative dendritic analysis of Wernicke's area in humans. I. Lifespan changes. J. Comp. Neurol. 327, 83–96 10.1002/cne.903270107 8432909

[B63] Jacobs B Scheibel A. B. (2002). Regional dendritic variation in primate cortical pyramidal cells, in Cortical Areas: Unity and Diversity (Conceptual Advances in Brain Research Series), eds Schüz A. Miller R. (London: Taylor and Francis), 111–131

[B64] Kaas J. H. (2008). The evolution of the complex sensory and motor systems of the human brain. Brain Res. Bull. 75, 384–390 10.1016/j.brainresbull.2007.10.009 18331903 PMC2349093

[B65] Lainé J. Axelrad H. (1994). The candelabrum cell: a new interneuron in the cerebellar cortex. J. Comp. Neurol. 339, 159–173 10.1002/cne.903390202 8300903

[B66] Lainé J. Axelrad H. (1996). Morphology of the Golgi-Impregnated Lugaro cell in the rat cerebellar cortex: a reappraisal with a description of its axon. J. Comp. Neurol. 375, 618–640 8930789 10.1002/(SICI)1096-9861(19961125)375:4<618::AID-CNE5>3.0.CO;2-4

[B67] Lainé J. Axelrad H. (2002). Extending the cerebellar Lugaro cell class. Neuroscience 115, 363–374 10.1016/S0306-4522(02)00421-9 12421603

[B68] Landau E. (1933). La cellule synarmotique dans le cervelet humain. Arch. Anat. 17, 273–285

[B69] Lange W. (1975). Cell number and cell density in the cerebellar cortex of man and some other mammals. Cell Tissue Res. 157, 115–124 10.1007/BF00223234 804353

[B70] Larsell O. (1970). The Comparative Anatomy and Histology of the Cerebellum from Monotremes through Apes. Minneapolis, MN: The University of Minnesota

[B71] Larsell O. Jansen J. (1972). The Comparative Anatomy and Histology of the Cerebellum: the Human Cerebellum, Cerebellar Connections, and Cerebellar Cortex. Minneapolis, MN: The University of Minnesota

[B72] Leto K. Carletti B. Williams I. M. Magrassi L. Rossi F. (2006). Different types of cerebellar GABAergic interneurons originate from a common pool of multipotent progenitor cells. J. Neurosci. 26, 11682–11694 10.1523/JNEUROSCI.3656-06.2006 17093090 PMC6674781

[B73] Lu D. He L. Xiang W. Ai W.-M. Cao Y. Wang X.-S. (2013). Somal and dendritic development of human CA3 pyramidal neurons from midgestation to middle childhood: a quantitative Golgi study. Anat. Rec. 296, 123–132 10.1002/ar.22616 23152308

[B74] Lugaro E. (1894). Sulle connessioni tra gli elementi nervosi della corteccia cerebellare con considerazioni generali sul significato fisiologico dei rapporti tra gli elementi nervosi. Riv. Sper. Fren. Med. Leg. 20, 297–331

[B75] MacLeod C. E. Zilles K. Schleicher A. Rilling J. K. Gibson K. R. (2003). Expansion of the neocerebellum in Hominoidea. J. Hum. Evol. 44, 401–429 10.1016/S0047-2484(03)00028-9 12727461

[B76] Manger P. R. Pillay P. Madeko B. C. Bhagwandin A. Gravett N. Moon D.-J. (2009). Acquisition of brains from the African elephant (Loxodonta africana): perfusion-fixation and dissection. J. Neurosci. Methods 179, 16–21 10.1016/jneumeth.2009.01.001 19168095

[B77] Manger P. R. Cort J. Ebrahim N. Goodman A. Henning J. Karolia M. (2008). Is 21st century neuroscience too focused on the rat/mouse model of the brain function and dysfunction? Front. Neuroanat. 2:5 10.3389/neuro.05.005.2008 19127284 PMC2605402

[B78] Manger P. R. Spocter M. A. Patzke N. (2013). The evolutions of large brain size in mammals: the “over-700-gram club quartet.” Brain Behav. Evol. 82, 68–78 10.1159/000352056 23979457

[B79] Marr D. (1969). A theory of cerebellar cortex. J. Physiol. 202, 437–470 5784296 10.1113/jphysiol.1969.sp008820 PMC1351491

[B80] Marino L. Rilling J. K. Lin S. K. Ridgway S. H. (2000). Relative volume of the cerebellum in dolphins and comparison with anthropoid primates. Brain Behav. Evol. 56, 204–211 10.1159/000047205 11154999

[B81] Marshall C. D. Clark L. A. Reep R. L. (1998). The muscular hydrostat of the Florida manatee (*Trichechus manatus latirostris*): a functional morphological model of perioral bristle use. Mar. Mamm. Sci. 14, 290–303 10.111/j.1748-7692.1998.tb00717.x

[B82] Marshall C. D. Maeda H. Iwata M. Furuta M. Asano S. Rosas F. (2003). Orofacial morphology and feeding behaviour of the dugong, Amazonian, West African and Antillean manatees (Mammalia: Sirenia): functional morphology of the muscular-vibrissal complex. J. Zool. Lond. 259, 245–260 10.1017/S09528369022003205

[B83] Maseko B. C. Jacobs B. Spocter M. A. Sherwood C. C. Hof P. R. Manger P. R. (2012a). Qualitative and quantitative aspects of the microanatomy of the African elephant cerebellar cortex. Brain Behav. Evol. 81, 40–55 10.1159/000345565 23296570

[B84] Maseko B. C. Spocter M. A. Haagensen M. M. Manger P. R. (2012b). Elephants have relatively the largest cerebellum size of mammals. Anat. Rec. 295, 661–672 10.1002/ar.22425 22282440

[B85] Mavroudis I. A. Manani M. G. Petrides F. Petsoglou K. Njau S. D. Costa V. G. (2013). Dendritic and spinal pathology of the Purkinje cells from the human cerebellar vermis in Alzheimer's disease. Psychiat. Danub. 25, 221–226 24048388

[B86] Meek J. (1992). Comparative aspects of cerebellar organization: from mormyrids to mammals. Eur. J. Morphol. 30, 37–51 1642952

[B87] Meek J. Nieuwenhuys R. (1991). Palisade pattern of mormyrid Purkinje cells: a correlated light and electron microscopic study. J. Comp. Neurol. 306, 156–192 10.1002/cne.903060111 2040726

[B88] Meek J. Yang J. Y. Han V. Z. Bell C. C. (2008). Morphological analysis of the mormyrid cerebellum using immunohistochemistry, with emphasis on the unusual neuronal organization of the valvula. J. Comp. Neurol. 510, 396–421 10.1002/cne.21809 18663756 PMC5862061

[B89] Melik-Musyan A. B. Fanardzhyan V. V. (1998). Histological identification of Lugaro cells in the cat cerebellum. Neurosci. Behav. Physiol. 28, 486–489 10.1007/BF02463006 9809285

[B90] Melik-Musyan A. B. Fanardzhyan V. V. (2004). Morphological characteristics of Lugaro cells in the cerebellar cortex. Neurosci. Behav. Physiol. 34, 633–638 10.1023/B:NEAB.0000028297.30474.f9 15368913

[B91] Mercado E. 3rd. Schneider J. N. Pack A. A. Herman L. M. (2010). Sound production by singing humpback whales. J. Acoust. Soc. Am. 178, 2678–2691 10.1121/1.3309453 20370048

[B92] Milatovic D. Montine T. J. Zaja-Milatovic S. Madison J. L. Bowman A. B. Aschner M. (2010). Morphometric analysis in neurogenerative disorders. Curr. Protoc. Toxicol. 12, 1–14 10.1002/0471140856.tx1216s43 20401325 PMC2855147

[B93] Murakami T. Morita Y. (1987). Morphology and distribution of the projection neurons in the cerebellum in a Teleost, *Sebastiscus marmoratus*. J. Comp. Neurol. 256, 607–623 10.1002/cne.902560413 3558892

[B95] Nicholson C. Llinas R. (1971). Field potentials in the alligator cerebellum and theory of their relationship to Purkinje cell dendritic spikes. J. Neurophysiol. 34, 509–531 4329777 10.1152/jn.1971.34.4.509

[B96] Nieuwenhuys R. Nicholson C. (1967). The cerebellum of mormyrids. Nature 215, 764–765 10.1038/215764a0 4168449

[B97] Norris K. S. Prescott J. H. Asa-Dorian P. V. Perkins P. (1961). An experimental demonstration of echo-location behavior in the porpoise, *Tursiops truncatus* (Montagu). Biol. Bull. 120, 163–176 10.2307/1539374

[B98] Ohm T. G. Diekmann S. (1994). The use of Lucifer Yellow and Mini-Ruby for intracellular staining in fixed brain tissue: methodological considerations evaluated in rat and human autopsy brains. J. Neurosci. Methods 55, 105–110 10.1016/0165-0270(94)90046-9 7534362

[B99] O'Leary J. L. Petty J. Smith J. M. O'Leary M. Inukai J. (1968). Cerebellar cortex of rat and other animals: a structural and ultrastructural study. J. Comp. Neurol. 134, 401–432 4181063 10.1002/cne.901340404

[B100] Onodera S. Hicks P. T. (1999). Review: evolution of the motor system: why the elephant's trunk works like a human's hand. Neuroscientist 5, 217–226 10.1177/107385849900500411

[B101] Palay S. L. Chan-Palay V. (1974). Cerebellar Cortex: Cytology and Organization. Berlin: Springer-Verlag 10.1007/978-3-642-65581-4

[B102] Parsons L. M. Bower J. M. Gao J.-H. Xiong J. Li J. Fox P. T. (1997). Lateral cerebellar hemispheres actively support sensory acquisition and discrimination rather than motor control. Learn. Mem. 4, 49–62 10.1101/lm.4.1.49 10456053

[B104] Rakic P. (1972). Extrinsic cytological determinants of basket and stellate cell dendritic pattern in the cerebellar molecular layer. J. Comp. Neurol. 146, 335–354 10.1002/cne.901460304 4628749

[B105] Ramón y Cajal S. (1909, 1911). Histologie du Système Nerveux de l'Homme et des Vertébrés. Azoulay L. (transl.) Paris: Maloine

[B106] Rapp M. Segev I. Yarom Y. (1994). Physiology, morphology and detailed passive models of guinea-pig cerebellar Purkinje cells. J. Physiol. 474, 101–118 8014888 10.1113/jphysiol.1994.sp020006 PMC1160299

[B107] Rasmussen L. E. L. Munger B. L. (1996). The sensorineural specializations of the trunk tip (finger) of the Asian elephant, *Elephas maximus*. Anat. Rec. 246, 127–134 10.1002/(SICI)1097-0185(199609)246:1<127::AID-AR14>3.0.CO;2-R 8876831

[B108] Reep R. L. O'Shea T. J. (1990). Regional brain morphometry and lissencephaly in the Sirenia. Brain Behav. Evol. 35, 185–194 10.1159/000115866 2379080

[B109] Reep R. L. Stoll M. L. Marshall C. D. Homer B. L. Samuelson D. A. (2001). Microanatomy of facial vibrissae in the Florida manatee: the basis for specialized sensory function and oripulation. Brain Behav. Evol. 58, 1–14 10.1159/000047257 11799274

[B110] Rilling J. K. (2006). Human and nonhman primate brains: are they allometrically scaled versions of the same design? Evol. Anthropol. 15, 65–77 10.1002/evan.20095

[B111] Rilling J. K. Insel T. R. (1998). Evolution of the cerebellum in primates: differences in relative volume among monkeys, apes and humans. Brain Behav. Evol. 52, 308–314 10.1159/000006575 9807015

[B112] Roitman M. F. Na E. Anderson G. Jones T. A. Bernstein I. L. (2002). Induction of a salt appetite alters dendritic morphology in nucleus accumbens and sensitizes rats to amphetamine. J. Neurosci. 22, RC225 (1–5). 12040084 10.1523/JNEUROSCI.22-11-j0001.2002 PMC6758808

[B113] Roth A. Häuser M. (2001). Compartmental models of rat cerebellar Purkinje cells based on simultaneous somatic and dendritic patch-clamp recordings. J. Physiol. 535, 445–472 10.1111/j.1469-7793.2001.00445.x 11533136 PMC2278793

[B114] Sahin M. Hockfield S. (1990). Molecular identification of the Lugaro cell in the cat cerebellar cortex. J. Comp Neurol. 301, 575–584 2273099 10.1002/cne.903010407

[B115] Sarko D. K. Catania K. C. Leitch D. B. Kaas J. H. Herculano-Houzel S. (2009). Cellular scaling rules of insectivore brains. Front. Neuroanat. 3:8 10.3389/neuro.05.008.2009 19636383 PMC2713736

[B116] Sawada Y. Kajiwara G. Iizuka A. Takayama K. Shuvaev A. N. Koyama C. (2010). High transgene expression by lentiviral vectors causes maldevelopment of Purkinje cells *in vivo*. Cerebellum 9, 291–302 10.1007/s12311-010-0161-1 20178014

[B117] Schadé J. P. Caveness W. F. (1968). IV. Alteration in dendritic organization. Brain Res. 7, 59–86 10379825

[B118] Scheibel M. E. Scheibel A. B. (1978). The methods of Golgi, in Neuroanatomical Research Techniques, ed Robertson R. T. (New York, NY: Academic Press), 89–114 10.1016/B978-0-12-590350-9.50011-2

[B119] Schilling K. Oberdick J. Rossi R. Baader S. L. (2009). Besides Purkinje cells and granule neurons: an appraisal of the cell biology of the interneurons of the cerebellar cortex. Histochem. Cell Biol. 130, 601–615 10.1007/s00418-008-0483-y 18677503

[B120] Simat M. Parpan F. Fritschy J.-M. (2007). Heterogeneity of glycinergic and gabaergic interneurons in the granule cell layer of mouse cerebellum. J. Comp. Neurol. 500, 71–83 10.1002/cne.21142 17099896

[B121] Sholl D. A. (1953). Dendritic organization of the neurons of the visual and motor cortices of the cat. J. Anat. 87, 387–406 13117757 PMC1244622

[B122] Shoshani J. Kupsky W. J. Marchant G. H. (2006). Elephant brain part I: gross morphology, functions, comparative anatomy, and evolution. Brain Res. Bull. 70, 124–157 10.1016/j.brainresbull.2006.03.016 16782503

[B123] Smaers J. B. Steele J. Zilles K. (2011). Modeling the evolution of cortico-cerebellar systems in primates. Ann. N.Y. Acad. Sci. 1225, 176–190 10.1111/j.1749-6632.2011.06003.x 21535004

[B124] Soltis J. (2009). Vocal communication in African elephants (*Loxodonta africana*). Zoo Biol. 28, 1–18 10.1002/zoo.20251 19434672

[B125] Sultan F. Bower J. M. (1998). Quantitative Golgi study of the rat cerebellar molecular layer interneurons using principal component analysis. J. Comp. Neurol. 393, 353–373 10.1002/(SICI)1096-9861(19980413)393:33.3.CO;2-7 9548555

[B126] Sultan F. Braitenberg V. (1993). Shapes and sizes of different mammalian cerebella. A study in quantitative comparative neuroanatomy. J. Hirnforsch. 1, 79–92 8376757

[B127] Sultan F. Glickstein M. (2007). The cerebellum: comparative and animal studies. Cerebellum 6, 168–176 10.1080/14734220701332486 17786812

[B128] Surlyke A. Ghose K. Moss C. F. (2009). Acoustic scanning of natural scenes by echolocation in the big brown bat, *Eptesicus fuscus*. J. Exp. Biol. 212, 1011–1020 10.1242/jeb.024620 19282498 PMC2726860

[B129] Ulfhake B. (1984). A morphometric study of the soma, first-order dendrites and proximal axon of cat lumbar α-motoneurones intracellularly labeled with HRP. Exp. Brain Res. 56, 327–334 10.1007/BF00236288 6479266

[B130] Uylings H. B. M. Ruiz-Marcos A. van Pelt J. (1986). The metric analysis of three-dimensional dendritic tree patterns: a methodological review. J. Neurosci. Methods 18, 127–151 10.1016/0165-0270(86)90116-0 3540466

[B131] Welker W. (1964). Analysis of the sniffing behavior of the albino rat. Behavior 22, 223–244 10.1163/156853964X00030

[B132] Wen Q. Stepanyants A. Elston G. N. Grosberg A. Y. Chklovskii D. B. (2009). Maximization of the connectivity repertoire as a statistical principle governing the shapes of dendritic arbors. Proc. Natl. Acad. Sci. U.S.A. 106, 12536–12541 10.1073/pnas.0901530106 19622738 PMC2713752

[B133] Williams M. N. Grajales C. A. G. Kurkiewicz D. (2013). Assumptions of multiple regression: correcting two misconceptions. Pract. Assess. Res. Eval. 18 Available online at: http://pareonline.net/getvn.asp?v=18&n=11

[B134] Williams R. S. Ferrante R. J. Caviness V. S. Jr. (1978). The Golgi rapid method in clinical neuropathology: morphological consequences of suboptimal fixation. J. Neuropath. Exp. Neurol. 37, 13–33 10.1097/00005072-197801000-00002 73572

[B135] Wu K.-Y. Zhou X.-P. Luo Z.-G. (2010). Geranylgeranyltransferase I is essential for dendritic development of cerebellar Purkinje cells. Mol. Brain 3:18 10.1186/1756-6606-3-18 20540740 PMC2902468

