# Corrigendum: Comparative neuronal morphology of the cerebellar cortex in afrotherians, carnivores, cetartiodactyls, and primates

**DOI:** 10.3389/fnana.2014.00069

**Published:** 2014-07-23

**Authors:** Bob Jacobs, Nicholas L. Johnson, Devin Wahl, Matthew Schall, Busisiwe C. Maseko, Albert Lewandowski, Mary A. Raghanti, Bridget Wicinski, Camilla Butti, William D. Hopkins, Mads F. Bertelsen, Timothy Walsh, John R. Roberts, Roger L. Reep, Patrick R. Hof, Chet C. Sherwood, Paul R. Manger

**Affiliations:** ^1^Laboratory of Quantitative Neuromorphology, Psychology, Colorado CollegeColorado Springs, CO, USA; ^2^Faculty of Health Sciences, School of Anatomical Sciences, University of the WitwatersrandJohannesburg, South Africa; ^3^Cleveland Metroparks ZooCleveland, OH, USA; ^4^Department of Anthropology, Kent State UniversityKent, OH, USA; ^5^Fishberg Department of Neuroscience and Friedman Brain Institute, Icahn School of Medicine at Mount SinaiNew York, NY, USA; ^6^Division of Developmental and Cognitive Neuroscience, Yerkes National Primate Research CenterAtlanta, GA, USA; ^7^Center for Zoo and Wild Animal HealthCopenhagen Zoo, Frederiksberg, Denmark; ^8^Smithsonian National Zoological ParkWashington, DC, USA; ^9^Department of Physiological Sciences, University of FloridaGainesville, FL, USA; ^10^Department of Anthropology, The George Washington UniversityWashington, DC, USA

**Keywords:** dendrite, morphometry, Golgi method, brain evolution, cerebellum

There was a typographical error in Table 2 of Jacobs et al. (2014) as one partial row of data is repeated. Specifically, for Vol., the values for the Clouded Leopard Stellate cells are repeated in place of the Granule cells. For the Clouded Leopard Granule cells, the values should NOT be the following:

Mean = 691.9, *SD* = 222.2, L95% = 247.4, U95% = 1136.4.

Instead, these values should be:

Mean = 51.4, *SD* = 32.9, L95% = −14.4, U95% = 117.3.

None of the corrections are in bold font.

**Table 2:**
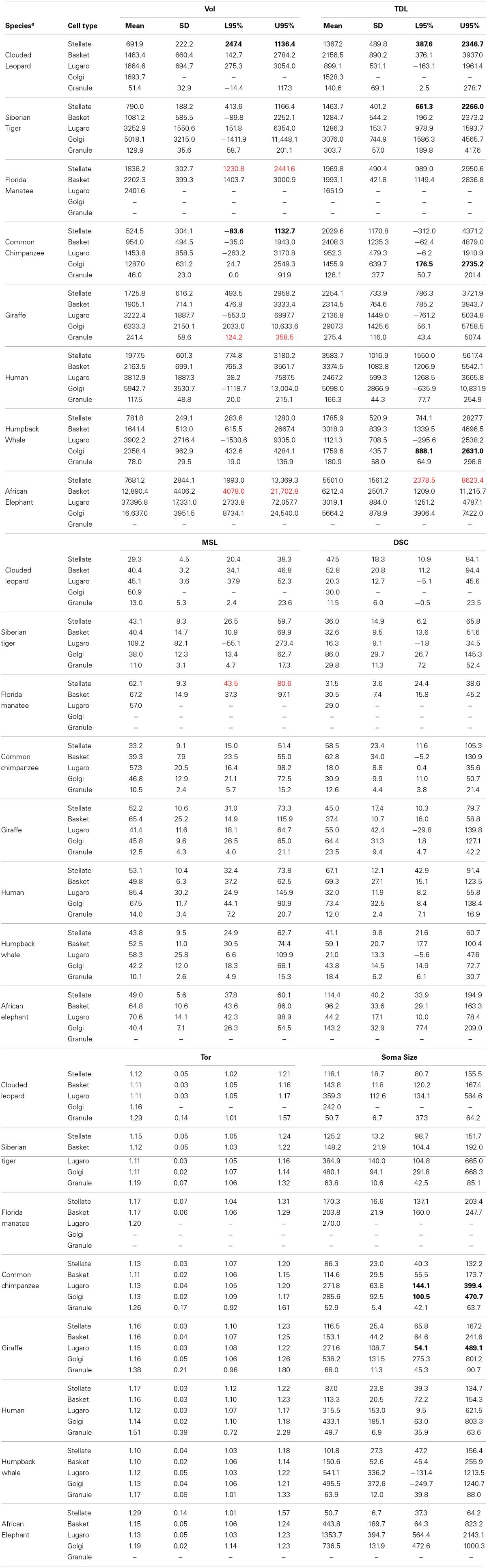
**Dendritic measures and soma size for each neuronal type across species^a^**.

*^a^ Dependent measures are: Volume (Vol, μm^3^), Total Dendritic Length (TDL, μm), Mean Segment Length (MSL), Dendritic Segment Count (DSC), Tortuosity (Tor), and Soma Size (μm^2^). Species are arranged in from smallest (clouded leopard) to largest (African elephant) in terms of brain mass. L95% = lower95% confidence interval; U95% = upper95% confidence interval. **Bold numbers** represent comparisons that were significantly different from the elephant values; red numbers represent comparisons that were significantly different from the clouded leopard values. See text for further explanation*.

## Conflict of interest statement

The authors declare that the research was conducted in the absence of any commercial or financial relationships that could be construed as a potential conflict of interest.

